# Extracellular Actin Is a Receptor for *Mycoplasma hyopneumoniae*

**DOI:** 10.3389/fcimb.2018.00054

**Published:** 2018-02-27

**Authors:** Benjamin B. A. Raymond, Ranya Madhkoor, Ina Schleicher, Cord C. Uphoff, Lynne Turnbull, Cynthia B. Whitchurch, Manfred Rohde, Matthew P. Padula, Steven P. Djordjevic

**Affiliations:** ^1^The ithree Institute, Faculty of Science, University of Technology Sydney, Ultimo, NSW, Australia; ^2^Central Facility for Microscopy, Helmholtz Centre for Infection Research, Braunschweig, Germany; ^3^Leibniz-Institute DSMZ-German Collection of Microorganisms and Cell Cultures, Braunschweig, Germany; ^4^Proteomics Core Facility, University of Technology, Sydney, NSW, Australia

**Keywords:** extracellular actin, mycoplasma infections, host-pathogen interactions, bacterial adhesins, super resolution microscopy

## Abstract

*Mycoplasma hyopneumoniae*, an agriculturally important porcine pathogen, disrupts the mucociliary escalator causing ciliostasis, loss of cilial function, and epithelial cell death within the porcine lung. Losses to swine production due to growth rate retardation and reduced feed conversion efficiency are severe, and antibiotics are used heavily to control mycoplasmal pneumonia. Notably, little is known about the repertoire of host receptors that *M. hyopneumoniae* targets to facilitate colonization. Here we show, for the first time, that actin exists extracellularly on porcine epithelial monolayers (PK-15) using surface biotinylation and 3D-Structured Illumination Microscopy (3D-SIM), and that *M. hyopneumoniae* binds to the extracellular β-actin exposed on the surface of these cells. Consistent with this hypothesis we show: (i) monoclonal antibodies that target β-actin significantly block the ability of *M. hyopneumoniae* to adhere and colonize PK-15 cells; (ii) microtiter plate binding assays show that *M. hyopneumoniae* cells bind to monomeric G-actin in a dose dependent manner; (iii) more than 100 *M. hyopneumoniae* proteins were recovered from affinity-chromatography experiments using immobilized actin as bait; and (iv) biotinylated monomeric actin binds directly to *M. hyopneumoniae* proteins in ligand blotting studies. Specifically, we show that the P97 cilium adhesin possesses at least two distinct actin-binding regions, and binds monomeric actin with nanomolar affinity. Taken together, these observations suggest that actin may be an important receptor for *M. hyopneumoniae* within the swine lung and will aid in the future development of intervention strategies against this devastating pathogen. Furthermore, our observations have wider implications for extracellular actin as an important bacterial receptor.

## Introduction

*Mycoplasma hyopneumoniae*, the etiological agent of porcine enzootic pneumonia (PEP; Mare and Switzer, 1965; Goodwin et al., 1967), remains one of the most economically significant pathogens that causes reduced growth rate along with lower feed conversion efficiency in swine. Attributing losses to pork production incurred by *M. hyopneumoniae* are complicated by polymicrobial infections but recent estimates for US production systems are in the order of $US10 per head (Holst et al., 2015). This is significantly more than US estimates of $200 million to $1 billion p.a. reported earlier (Clark et al., 1993). On top of this, these estimates fail to account for the environmental impact caused by the release of multiple antibiotic resistant bacterial populations and significant quantities of unmetabolized antimicrobials into effluent ponds, which are in turn used to fertilize agricultural land in many parts of the world, particularly in China; the largest producer and consumer of pork (Wyrsch et al., 2015; Zhu et al., 2015). *M. hyopneumoniae* has an affinity for receptors on the surface of cilia that line the epithelium in the upper respiratory tract of pigs and destroys the mucociliary escalator creating a favorable environment for secondary bacterial infections (Ciprián et al., 1988; Caruso and Ross, 1990; Marois et al., 2007). *M. hyopneumoniae* can also potentiate disease caused by porcine reproductive and respiratory syndrome virus (PRRSV), swine influenza virus (SIV), and porcine circovirus type 2 (PCV2) (Thacker et al., 2000, 2001; Pallarés et al., 2002; Opriessnig et al., 2004). Tetracyclines, macrolides, lincosamides, fluoroquinolones, and aminoglycosides are used widely to treat disease caused by *M. hyopneumoniae* but a more diverse combination of antibiotics is used to prevent polymicrobial respiratory infections in pigs (Stipkovits et al., 2001; Maes et al., 2008). Consequently, disease caused by *M. hyopneumoniae* is one of the major drivers of antibiotic consumption in swine production globally. Thus, there is a pressing need to develop alternatives to antimicrobials to control pathogens that inflict major economic losses in intensively-reared food animals.

Studies over the past 15 years have focussed on understanding how *M. hyopneumoniae* attaches to cilia and colonizes the respiratory epithelium. The P97 and P102 family of multifunctional cilium adhesins are highly expressed (Pendarvis et al., 2014) on the surface of *M. hyopneumoniae* as cleavage fragments that bind several host molecules including highly sulfated glycosaminoglycans (GAGs), fibronectin, and plasminogen (Burnett et al., 2006; Wilton et al., 2009; Deutscher et al., 2010; Seymour et al., 2010, 2011, 2012; Bogema et al., 2011, 2012; Raymond et al., 2013, 2015; Tacchi et al., 2014, 2016). GAGs decorate the surface of cilia within the swine respiratory tract (Erlinger, 1995) and are primary receptors for adhesins on the surface of *M. hyopneumoniae* (Burnett et al., 2006; Jenkins et al., 2006; Wilton et al., 2009; Deutscher et al., 2010; Seymour et al., 2010, 2011, 2012; Bogema et al., 2011, 2012; Raymond et al., 2013, 2015; Tacchi et al., 2014; Jarocki et al., 2015). Upon colonization, *M. hyopneumoniae* induces ciliostasis, loss of cilia, and epithelial cell death (DeBey and Ross, 1994) but it is unknown if *M. hyopneumoniae* can colonize after these events. Furthermore, the identities of other cell surface receptors, especially after cilial removal, that are targeted by *M. hyopneumoniae* are poorly understood. One such receptor that is of particular interest is the major cytoskeletal protein actin that has been shown to be bound by several bacterial pathogens such as group B streptococcus, *Mycoplasma suis*, and *Legionella pneumophila* (Boone and Tyrrell, 2012; Bugalhão et al., 2015; Zhang et al., 2015). Actin is potentially underappreciated as a bacterial receptor and is reported to be expressed on the surface of a wide range of eukaryote cells (Chen et al., 1978; Owen et al., 1978; Jones et al., 1979; Sanders and Craig, 1983; Rosenblatt et al., 1985a; Bach et al., 1986; Pardridge et al., 1989; Por et al., 1991; Dudani and Ganz, 1996; Smalheiser, 1996; Miles et al., 2006; Sandiford et al., 2015; Fu et al., 2017; Sudakov et al., 2017). Studies that seek to investigate the ability of *M. hyopneumoniae* to bind to actin have not been undertaken.

Porcine kidney epithelial-like (PK-15) cells were established as a cell line to study the *in vitro* adherence of *M. hyopneumoniae* (Zielinski et al., 1990; Burnett et al., 2006; Wilton et al., 2009; Deutscher et al., 2010; Seymour et al., 2010, 2011). Here, using 3D-structured illumination microscopy (3D-SIM), we show *M. hyopneumoniae* binds to a subset of actin filaments that are expressed on the surface of porcine kidney epithelial cells (PK-15 cells). We assessed the ability of *M. hyopneumoniae* proteins to bind actin using microtiter plate binding assays, affinity chromatography, microscale thermophoresis, immunofluorescence microscopy, and ligand blotting. We also examined the ability of monoclonal antibodies that target β-actin (mAb_β-*act*_) to compete for actin-binding sites on the surface of *M. hyopneumoniae*.

## Methods and materials

### Bacterial strains and culture

*Mycoplasma hyopneumoniae* strain 232 was grown in modified Friis medium (Scarman et al., 1997) as previously described (Bereiter et al., 1990). Broth cultures were typically incubated at 37°C for ~16 h until the medium turned yellow. For growth on solid agar, cells were plated onto Friis agar as previously described (Kobisch and Friis, 1996).

### Protein expression, purification, and generation of antisera

Purified P97_232_ fragments were expressed and purified as previously described (Raymond et al., 2015). Antiserum against F2_P94−J_ was generated in a previous study and with titers determined empirically (Jenkins et al., 2006).

### Quantitative adherence and inhibition assays

Porcine kidney epithelial-like monolayers (PK-15) were grown to semi-confluency and split into 12-well microtiter plates at ~10^5^ cells/ well and left to adhere overnight. For infection, an overnight *M. hyopneumoniae* culture of strain 232 was washed twice in PBS and resuspended in 25 mM HEPES in DMEM containing 5% fetal bovine serum (infection medium) and incubated at 37°C for 2 h. A proportion of these cells were diluted in fresh Friis broth and plated onto Friis agar to establish cell numbers prior to infection. *M. hyopneumoniae* cells were then added to each well containing PK-15 cells at a multiplicity of infection (MOI) of 0.85. *M. hyopneumoniae* cells were allowed to adhere for 16 h at 37°C/ 5% CO_2_. Non-adherent cells were removed by washing in PBS followed by incubating the wells in TrypLE™ (Thermo Fisher Scientific) for 20 min at 37°C to remove adherent PK-15 and *M. hyopneumoniae* cells. Cells were then removed by gently pipetting to create a relatively homogenous suspension followed by dilution in Friis broth to 10^−1^ and 10^−2^ and plating onto Friis agar. Plates were incubated for a minimum of 7 days at 37°C/ 5% CO_2_ prior to counting under a stereomicroscope. Inhibition experiments were performed by pre-incubating PK-15 cells with a 1:100 dilution of monoclonal antibodies against β-actin (IgG2A mAb_β-*act*_; ~5–20 μg ml^−1^ mAb, Sigma-Aldrich) for 2 h prior to the addition of *M. hyopneumoniae* cells. To ensure that IgG2A itself has no effect on the adherence of *M. hyopneumoniae* cells to PK-15 monolayers, isotype control experiments were performed. PK-15 monolayers, seeded onto coverslips as described below, were pre-incubated with either mAb_β-*act*_, murine IgG2A (Sigma-Aldrich), or no antibody for 2 h prior to infection with *M. hyopneumoniae*. Samples were then processed for immunofluorescence microscopy as described below. Slides were imaged using an Olympus BX51 Upright Epi Fluorescence Microscope at 20 × magnification and a constant 100 ms exposure, using the GFP filter to capture adherent *M. hyopneumoniae* cells labeled with CF™ 488. Five fields of view were captured per sample, equating to the adherence of *M. hyopneumoniae* cells across approximately 1,500 PK-15 cells per field of view. Image thresholding was performed using ImageJ and the mean fluorescence was calculated on the binary images generated. GraphPad Prism 7 was used to plot the data, including the standard error of the mean, and to perform the statistical analyses (unpaired *t-*test).

### Immunofluorescence microscopy of PK-15 monolayers infected with *M. hyopneumoniae* cells

Experiments were performed as described above except that PK-15 cells were split into microtiter plates containing glass coverslips (15 mm, 170 μm thickness; ProSciTech). Following infection, samples were washed 3 × in PBS and fixed in 2% methanol-free paraformaldehyde overnight at 4°C. Prior to commencing the following steps, samples were washed 3 × in PBS and all antibody dilutions were made in 1% BSA in PBS. Excess aldehydes were quenched in 100 mM glycine in PBS for 5 min at RT. Non-specific binding sites were blocked in 2% BSA/PBS for 1 h at RT. *M. hyopneumoniae* cells were labeled using polycloncal F2_P94−J_ rabbit antisera that recognizes the C-terminus of P97, incubated at a dilution of 1:500 for 1 h at RT. A 1:1,000 dilution of anti-rabbit CF™ 488- or 568-labeled secondary antibody (Biotium) was incubated for 1 h at RT. Cells were permeabilized in 0.5% (v/v) Triton X-100 in PBS for 5 min. DAPI (Roche) was then added for 5 min at RT to stain nucleic acids. Phalloidin conjugated to CF™ 568 (Biotium) was then added for 30 min at RT to stain filamentous (F-actin) actin. Coverslips were then mounted onto glass microscope slides in VECTASHIELD® (Vector Laboratories). Samples were imaged using a Nikon A1 Confocal Laser Scanning Microscope and a V3 DeltaVision OMX 3D-Structured Illumination Microscopy Imaging System (Applied Precision, GE Healthcare) as previously described (Strauss et al., 2012). Images were processed using Imaris Scientific 3D/4D image processing software (Bitplane AG, Zurich, Switzerland).

### Immunofluorescence microscopy of surface exposed actin in PK-15 monolayers

Experiments were performed identically to those described above except that a portion of PK-15 cells were not infected with *M. hyopneumoniae*. Surface actin was labeled prior to permeabilization using a murine monoclonal antibody against β-actin (mAb_β-*act*_, IgG2a isotype) at a dilution of 1:100 for 1 h at RT, followed by incubation with a 1:1,000 dilution of anti-murine CF™ 488-conjugated secondary antibodies (Biotium) for 1 h at RT. A number of control experiments were performed in order to confirm the specificity of mAb_β-*act*_. For surface exposed actin and to rule out the possibility of non-specific binding. Initially, an isotype control was performed using murine IgG2a that was diluted 1:100 in 2% BSA/PBS and incubated for 1 h at RT, followed by incubation with a 1:1,000 dilution of anti-murine CF™ 488-conjugated secondary antibodies (Biotium) for 1 h at RT. Secondary antibody controls were performed as described above, without primary antibody incubation. Additional controls were performed using mitochondria as an intracellular marker of permeabilization/compromization of the membrane. Uninfected PK-15 cells were fixed in paraformaldehyde as described above and were then either permeabilized in 0.5% Triton X-100 for 5 min or left unpermeabilized. Murine monoclonal antibodies against mitochondria (Abcam) were diluted 1:100 in 2% BSA/PBS and incubated overnight at 4°C, followed by incubation with a 1:1,000 dilution of anti-murine CF™ 488-conjugated secondary antibodies (Biotium) for 1 h at RT. Prior to mounting, all samples were stained with DAPI and imaged using an Olympus BX51 Upright Epi Fluorescence Microscope.

### Infection experiments for scanning electron microscopy

PK-15 cells were maintained in Dulbecco's modified eagle medium (DMEM, PAA Laboratories, Pasching, Austria) containing 10% fetal calf serum, 2 mM L-glutamine, 100 U ml^−1^ penicillin, and 0.1 mg ml^−1^ streptomycin in culture dishes (9 cm in diameter, TPP, Trasadingen, Switzerland) at 37°C in 5% CO_2_. Cells were harvested by trypsinization, resuspended in DMEM with 5% FCS and 2 mM L-glutamine and seeded into 24-well plates containing sterile 12 mm glass cover slips at 1 × 10^5^ cells/well. After growing overnight the medium was changed to infection medium (DMEM with 25 mM HEPES, 2 mM L-glutamine, 5% FCS) by adding 500 μl medium per well. 6 ml cultures of *M. hyopneumoniae* were harvested by centrifugation (14,000 rpm for 5 min, Eppendorf, Hamburg) and resuspended in 1 ml fresh infection medium for 2 h at 37°C on a roller, centrifuged again and resuspended in 120 μl infection medium. Ten to thirty microliters of the bacterial suspension were added per well and incubated at 37° in 5% CO_2_ for 4–48 h for infection. After different infection times samples were washed once in infection medium in a beaker and subsequently fixed with 2% glutaraldehyde and 5% formaldehyde in cacodylate buffer (100 mM sodium dimethylarsenic acid, 10 mM MgCl_2_, 10 mM CaCl_2_, 90 mM sucrose, pH 6.9) for 1 h on ice. Samples were then washed with TE buffer (20 mM TRIS, 2 mM EDTA, pH 7.0), dehydrated in a graded acetone series (10, 30, 50, 70, 90, 100%) for 10 min each step on ice and critical-point dried using liquid CO_2_. Samples were made conductive by sputter coating with a thin gold film. Samples were examined in a Zeiss field emission scanning electron microscope DSM982 Gemini at an acceleration voltage of 5 kV using the Everhart-Thornely SE-detector and the inlens SE-detector in a 50:50 ratio. Images were recorded on a MO-disk.

### Avidin purification of interacting proteins

The purification of porcine proteins from PK-15 monolayers that bind to proteins expressed on the surface of *M. hyopneumoniae* proteins was performed using methods described previously (Raymond et al., 2013). *M. hyopneumoniae* cells from a 250 ml culture were pelleted, washed, and resuspended in PBS containing 2 mM EZ-link Sulfo-NHS-Biotin for 30 s on ice. This ensured that *M. hyopneumoniae* cells did not lyse, and any remaining biotin molecules were quenched in 100 mM glycine for 10 min at RT. Cells were washed twice by centrifugation in PBS to remove excess glycine. Cells were gently lysed in 0.5% Triton X-100 in Tris-HCl pH 7.6, 150 mM NaCl with protease inhibitors on ice with vortexing and water bath sonication. Insoluble material was removed by centrifugation at 16,000 × *g* for 10 min. The cleared lysate was incubated with avidin agarose (Thermo Scientific) for 1 h at RT on a rotating wheel. The mixture was packed into a column and any unbound *M. hyopneumoniae* proteins were removed by thoroughly washing with PBS.

PK-15 cells were grown and harvested as previously described, without biotinylation (Raymond et al., 2013). The cleared protein lysate was incubated with the biotinylated-*M. hyopneumoniae*-avidin mixture overnight on a rotating wheel at 4°C. The mixture was packed into a column and the procedure was performed as previously described (Raymond et al., 2013).

### Biotinylation of PK-15 surface proteins

The biotinylation of PK-15 cells was performed as previously described with slight modifications (Raymond et al., 2013). PK-15 cells were grown to semi-confluency in a 175 cm^2^ flask, washed in PBS, and incubated with PBS containing 2 mM EZ-link Sulfo-NHS-Biotin for 30 s on ice. Excess biotin molecules were quenched in 100 mM glycine for 10 min at RT and cells were washed twice in PBS to remove excess glycine. Cells were gently lysed in 0.5% Triton X-100 in Tris-HCl pH 7.6, 150 mM NaCl with protease inhibitors on ice with vortexing and water bath sonication. Insoluble material was removed by centrifugation at 16,000 × *g* for 10 min. The cleared lysate was incubated with avidin agarose (Thermo Scientific) for 1 h at RT on a rotating wheel. The mixture was packed into a column and the washes, elutions, and preparations and identifications using LC-MS/MS were performed as previously described (Raymond et al., 2013).

### Preparation of monomerized actin

For binding experiments we utilized actin from bovine muscle (Sigma-Aldrich), likely α- or γ-actin. Sequence alignments performed on porcine α-, β-, and γ- isoforms demonstrated that they share 92.5% sequence identity (Supplementary Figure 5) and only differ substantially in the N-terminal 20 amino acids. This suggests that for the purposes of binding experiments, the utilization of actin from muscle (presumably α- and γ-actin) is sufficient to mimic β-actin binding. Additionally, bovine and porcine actin share 100% sequence identity, making it an appropriate substitution for investigating binding.

Actin was solubilized in 8 M Urea/ 20 mM Triethylammonium bicarbonate pH 8.0. Actin polymerization is dependent on disulfide bridges (Otterbein et al., 2001) and so in order to maintain actin in its monomerized form (G-actin), cysteine residues were reduced and alkylated in 20 mM acrylamide monomers and 5 mM Tributylphosphine for 90 min. Actin monomers were labeled in 20 × molar excess Sulfo-NHS-LC-Biotin for 3 h at RT. Removal of excess biotin and buffer exchange into PBS was performed in a PD-10 Desalting Column (GE Healthcare, Life Sciences) as per manufacturer instructions.

### Avidin purification of actin-binding proteins

Biotinylated actin was incubated with avidin agarose (Thermo Scientific) on a rotating wheel for 5 h. The slurry was packed into a column and the flow through collected. Unbound actin was thoroughly washed with PBS.

Native *M. hyopneumoniae* whole cell lysates were prepared as previously described (Raymond et al., 2013). The native lysate was incubated with the biotinylated actin-avidin agarose mixture overnight on a rotating wheel at 4°C. The mixture was packed into a column and the flow through collected. Unbound proteins were thoroughly washed and collected in PBS and interacting proteins were collected in 30% acetonitrile/ 0.4% trifluoroacetic acid. The elutions were concentrated in a 3,000 Da cutoff filter and acetone precipitated at −20°C for 30 min. Proteins were pelleted by centrifugation at 25,000 × *g* at 4°C for 30 min. Protein was resuspended in SDS sample buffer and separated by 1D SDS-PAGE. Proteins were in-gel trypsin digested and analyzed by LC–MS/MS.

### Whole cell ELISA

A 48 h Mhp232 culture was washed × 3 in PBS and fixed in 1% paraformaldehyde for 1 h at room temperature. Fixed cells were diluted in carbonate-coating buffer (18 mM NaHCO_3_, 27 mM Na_2_CO_3_; pH 9.5) and an equivalent of 100 μL of the original culture was added to each well of a MaxiSorp™ 96-well plate (Nunc®) and centrifuged at 2,000 rpm for 15 min. 20 mg ml^−1^ of BSA was used as a negative control for this experiment. Wells were washed × 3 in wash solution (0.2% tween-20 in PBS). Wells were blocked in 2% BSA in PBS for 1 h at room temperature. Wells were washed × 3 and incubated with 0, 1, 5, 10, 20, and 50 μg ml^−1^ of biotinylated bovine actin (Sigma-Aldrich) in 1% BSA in PBS for 1.5 h at room temperature. Wells were washed × 10 and incubated with a 1:2,000 dilution of ExtrAvidin®-Peroxidase (Sigma-Aldrich) in 1% BSA in PBS for 1 h at room temperature. Wells were washed × 10 and developed using TMB Substrate Reagent Set (BD Biosciences OptEIA™) and read at 650 nm after 15 and 30 min on a PowerWave HT Microplate Spectrophotometer (Bio-Tek). Background binding observed in wells without cells were subtracted from all readings. Data was analyzed using GraphPad Prism 6 software and has been plotted as the average of triplicate experiments.

### Microscale thermophoresis

Actin monomers were fluorescently labeled with amine reactive NT-647 dye (NanoTemper) in a three-fold molar excess for 30 min and excess dye was removed using a desalting column (NanoTemper). Tween-20 (0.5%) was added to prevent sample aggregation. Non-fluorescent P97 recombinant fragments F1_P97−232_, F3_P97−232_, and F4_P97−232_ were titrated against 100 nM fluorescent actin in a 1:1 serial dilution starting from 30 μM for F3_P97−232_ and F4_P97−232_ and 11 μM for F1_P97−232_ and samples were loaded into Monolith NT.115™ (NanoTemper) hydrophilic capillaries. Capillary scans were performed to confirm that no sample aggregation was occurring and that the fluorescence intensity was within the range of 80–1,500 counts. The data presented here was generated at 40% MST laser power measuring the thermophoretic movement after the MST laser is switched off for 30 s. NanoTemper analysis software was used for analysis and to generate *K*_*D*_ values.

### One- and two-dimensional gel electrophoresis and ligand blotting

1D and 2D SDS-PAGE was performed as previously described (Raymond et al., 2013) with minor modifications. Once *M. hyopneumoniae* proteins were transferred to PVDF membrane and blocked, biotinylated actin, or biotinylated PK-15 lysate prepared as previously described (Raymond et al., 2013) was incubated with the membrane overnight at 4°C. Membranes were probed with a 1:2,000 dilution of ExtrAvidin (Sigma-Aldrich) and developed with diaminobenzidine (Sigma-Aldrich).

### 1D LC–MS/MS using QTOF

Methods were performed as described previously with no modifications (Raymond et al., 2013).

### MS/MS data analysis

MS/MS data files were searched using Mascot as previously described (Raymond et al., 2013) with slight modifications. Data files from avidin purification of interacting proteins were searched for both bacterial and mammalian proteins in order to identify the biotinylated “bait” proteins eluted in their respective experiments.

## Results

### *M. hyopneumoniae* co-localizes with actin when adhering to PK-15 cells

**Figure 1:**
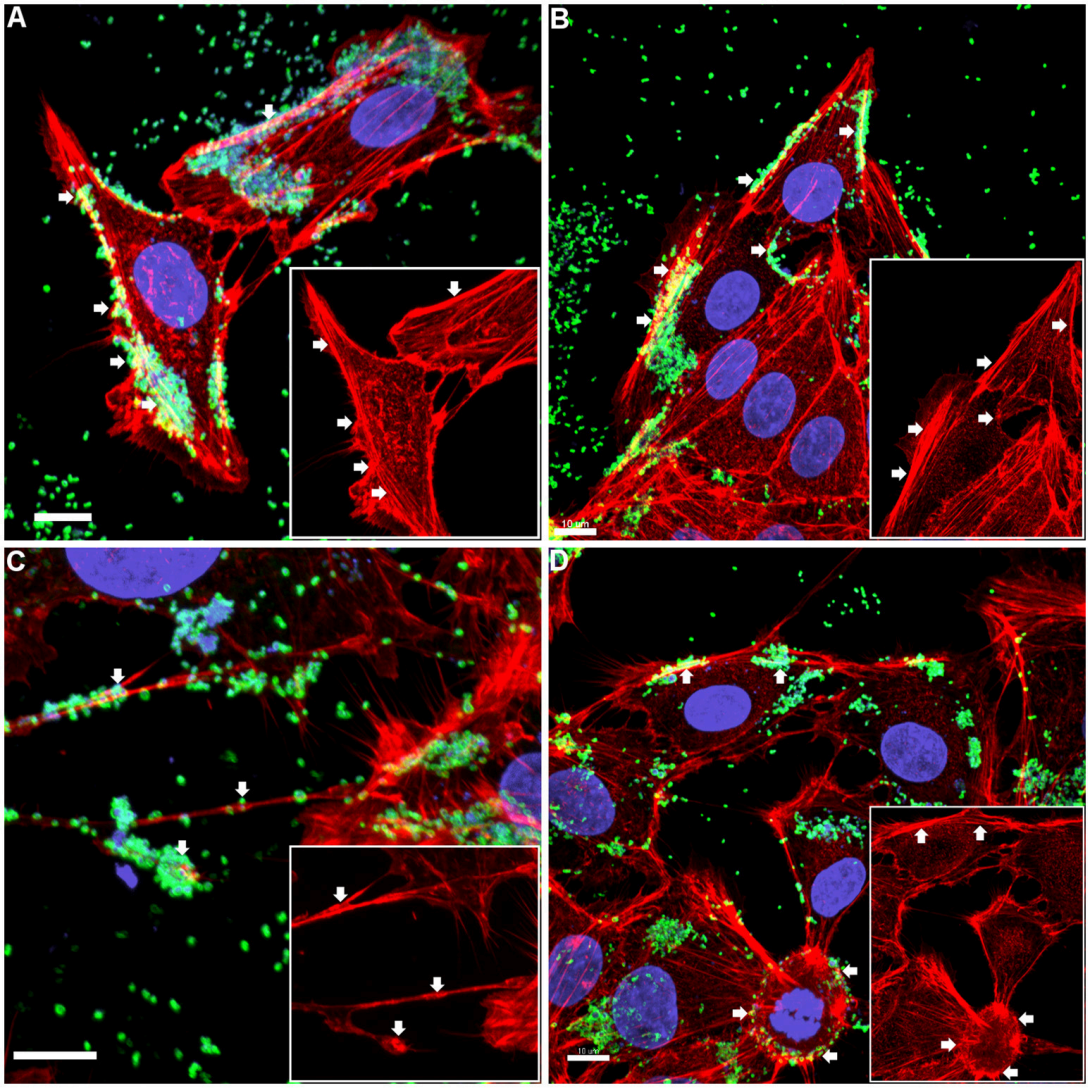
Confocal micrograph overlays of adherence patterns of *M. hyopneumoniae* cells to PK-15 monolayers. *M. hyopneumoniae* cells were labeled with F2_P94−J_ antisera conjugated to CF™ 488 (green), nucleic acids were stained with DAPI (blue), and F-actin was stained using phalloidin (red). *M. hyopneumoniae* cells can be seen adhering to the edges of the monolayer and to cellular projections that appear to be filopodia (white arrows). Inserts in each panel depict the phalloidin channel of each image (more specifically, the areas highlighted in the overlays), highlighting areas that stain intensely for F-actin (white arrows). **(A–D)** represents images taken from individual replicate experiments. Scale bars are 10 μm.

**Figure 2:**
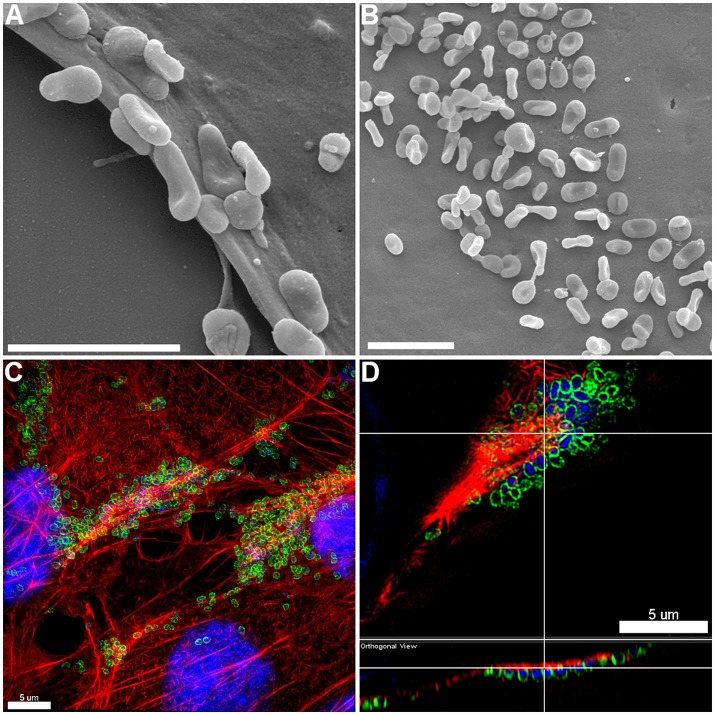
Adherence patterns of *M. hyopneumoniae* cells to PK-15 monolayers. **(A,B)** Scanning electron microscopy (SEM) image of *M. hyopneumoniae* cells adhering to PK-15 monolayers. *M. hyopneumoniae* cells favor the leading edge of epithelial cells **(A)** and select pockets on the top of the cells **(B)**, a pattern consistent with selective localization of epithelial receptors that are a target of adhesins on the surface of *M. hyopneumoniae*. Scale bars in **(A,B)** are 2 μm. **(C,D)** 3D-SIM images of *M. hyopneumoniae* adhering to PK-15 cell monolayers. *M. hyopneumoniae* cells were labeled with F2_P94−J_ antisera conjugated to CF™ 488 (green), nucleic acids were stained with DAPI (blue), and F-actin was stained using phalloidin (red). *M*. *hyopneumoniae* cells often localize to regions enriched in F-actin. **(D)** Orthogonal view showing *M. hyopneumoniae* cells associating closely with F-actin. Upper panel shows xy orientation and bottom panel shows xz orientation. White lines indicate the same area within the image.

The pattern of adherence of *M. hyopneumoniae* on PK-15 cell monolayers has not previously been studied by fluorescence microscopy. By using the filamentous actin (F-actin) dye phalloidin to stain the cytoskeleton of PK-15 cells and F2_P94−J_ antibodies that recognize the P97 adhesin, we repeatedly observed *M. hyopneumoniae* cells attaching to PK-15 cells along the leading edges as well as in discrete pockets on the surface that stained intensely with phalloidin (Figure 1 and Supplementary Figure 1). *M. hyopneumoniae* cells also co-localize with phalloidin-staining filaments that resemble filopodia (Figure 1C). Scanning Electron Microscopy images demonstrated a similar pattern of adherence, with *M. hyopneumoniae* cells preferentially colonizing the leading edges of the monolayer (Figures 2A,B). These observations suggest that *M. hyopneumoniae* cells can adhere to areas that stain with phalloidin; inferring that actin may be a potential receptor. 3D-SIM images of *M. hyopneumoniae* adhering to PK-15 cell monolayers once again demonstrated that regions staining intensely with phalloidin are favored (Figure 2C). A cross-sectional view of the image depicted in Figure 2C shows *M. hyopneumoniae* cells in close association with F-actin (Figure 2D). Given the intimate association between *M. hyopneumoniae* cells and actin, we hypothesized that *M. hyopneumoniae* cells may be targeting extracellular actin during adherence. However, PK-15 cell membranes must first be permeabilized to stain with phalloidin and it remains a challenge to determine if the F-actin making intimate contact with *M. hyopneumoniae* is extracellular.

### *M. hyopneumoniae* co-localizes with extracellular β-actin

There are three main isoforms of actin; α-, β-, and γ. α- and γ-actins are predominantly found in muscle while β-actin is the most predominant form found in non-muscle cells (Herman, 1993). Each of the actin isoforms exist either as a globular form (monomer), referred to as G-actin, or a polymerized filamentous form known as F-actin. Since our data suggests that *M. hyopneumoniae* co-localizes with F-actin (polymerized form of β-actin) we sought to investigate the localization of actin on the surface of uninfected PK-15 cells using 3D-SIM. To ensure that PK-15 cell monolayers did not become permeabilized during fixation, we labeled paraformaldehyde-fixed PK-15 cells that were permeabilized with Triton X-100 with a monoclonal antibody that recognizes mitochondria and compared the images with unpermeabilized cell preparations (no Triton X-100). Mitochondria were intensively stained in PK-15 cell preparations that were permeabilized using Triton X-100 (Supplementary Figure 2) but remained unlabeled (using identical exposure times) in cell preparations that were not treated with Triton X-100 (Supplementary Figure 2). These experiments indicated that the fixation protocol did not permeabilize PK-15 cell membranes. Additionally, isotype and secondary antibody controls confirmed that non-specific binding interactions were undetectable (Supplementary Figure 2).

**Figure 3:**
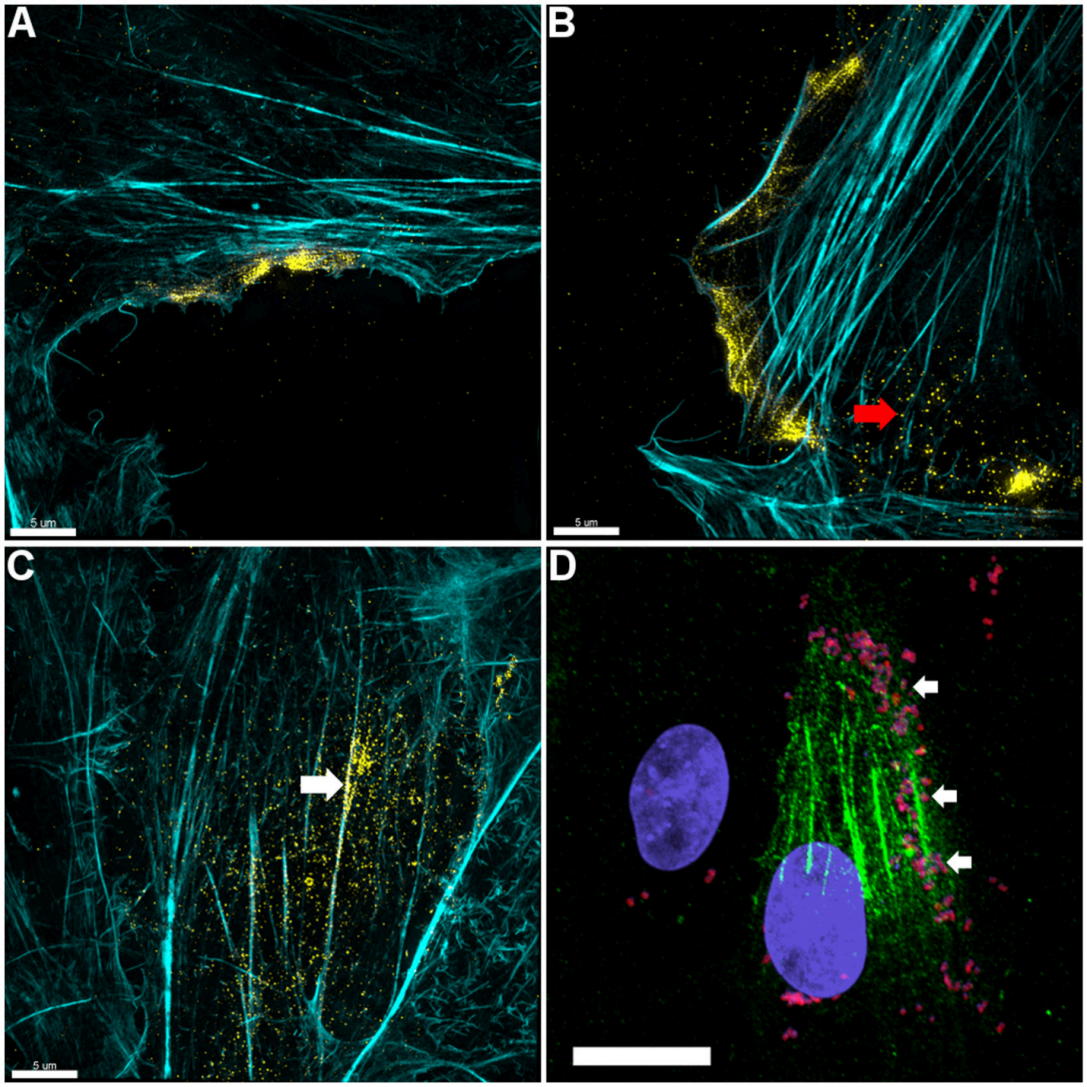
Actin is expressed on the surface of PK-15 cell monolayers. **(A–C)** Localization of actin on the surface of PK-15 cells. Uninfected monolayers were grown to semi-confluency, fixed in methanol-free paraformaldehyde, incubated with mAb_β−act_ prior to permeabilization, and conjugated to anti-murine CF™ 488 (yellow). F-actin was then stained using phalloidin (cyan) after permeabilization. Surface exposed actin can be seen accumulating at the leading edge **(A,B)** of cells in the monolayer as well as on the surface of cells along filaments that stained intensely with phalloidin **(C)**. Red arrow in **(B)** indicates what appears to be globular extracellular actin while the white arrow in **(C)** demonstrates extracellular actin that appears to colocalize with F-actin. **(D)** *M. hyopneumoniae* cells adhere on the surface of PK-15 cells in regions enriched with surface actin. *M. hyopneumoniae* cells (magenta colored cells indicated by white arrows) were labeled with F2_P94−J_ antisera conjugated to CF™ 568 (red) and in this case, surface actin appears green. The nuclei of *M. hyopneumoniae* and PK-15 cells were stained with DAPI (blue). Scale bars in **(A–C)** are 5 and 20 μm in **(D)**.

**Figure 4:**
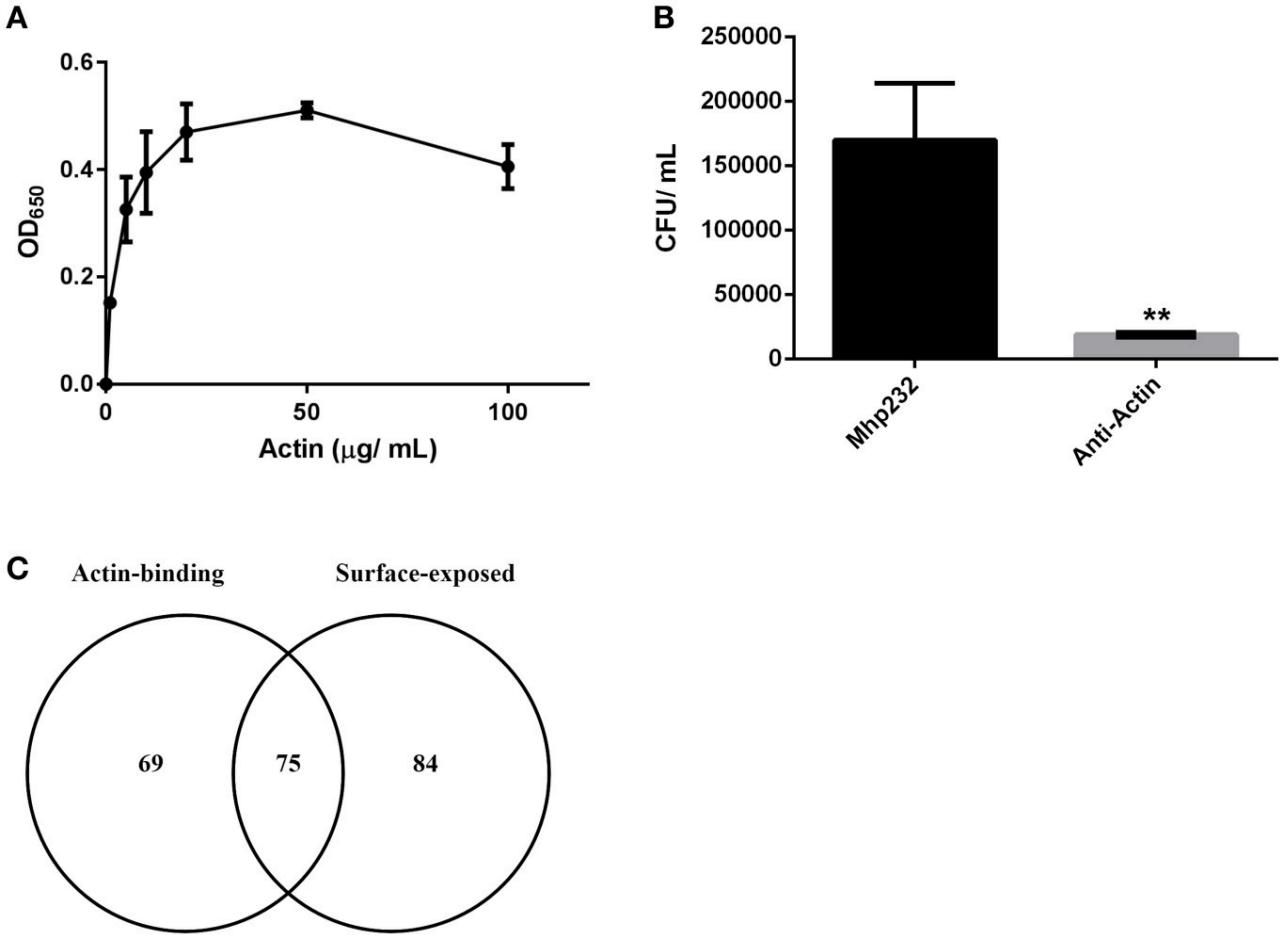
*M. hyopneumoniae* binds actin. **(A)** Microtiter plate binding assay of immobilized *M. hyopneumoniae* cells binding to actin in a dose-dependent and saturable manner. Each data point represents the average of individual triplicate experiments with the standard error of the mean shown. **(B)** Treatment of PK-15 cell monolayers with mAbs that target actin inhibited adherence of *M. hyopneumoniae* by ~90%. The data is presented as the average of triplicate experiments and the standard error of the mean. An unpaired *t*-test was performed with a *p* < 0.01, indicated as ^**^. **(C)** Venn diagram showing the overlap between putative actin-binding proteins purified using actin affinity chromatography, and *M. hyopneumoniae* surface proteins.

3D-SIM images of PK-15 cells that were labeled prior to permeabilization with mAb_β-*act*_ depict extracellular β-actin colocalizing with phalloidin-stained regions of the cell, including defined areas at the leading edge of PK-15 cells (Figures 3A–C). We also observed a pattern of staining where *M. hyopneumoniae* did not colocalize with phalloidin-staining filaments. In methodologically unrelated experiments, we identified tryptic peptides that mapped to β-actin from several discrete bands from an SDS-PAGE gel with molecular masses ranging from 40 kDa (predicted monomeric size of actin) to >100 kDa (polymerized forms of actin). The protein bands were recovered by avidin chromatography from a lysate of PK-15 cells whose surface proteins had been labeled with biotin (Supplementary Figure 3) indicating that extracellular actin exists in multiple forms on the surface of PK-15 cells. When we examined PK-15 cells that had been infected with *M. hyopneumoniae* under the same conditions, we observed the mycoplasma cells colocalizing with extracellular β-actin that was labeled with mAb_β-*act*_ (Figure 3D). Additionally, we could demonstrate mAb_β-*act*_ to compete with *M. hyopneumoniae* for surface accessible actin sites, reducing adherence by ~90% compared with non-treated controls (Figure 4B). Isotype control experiments demonstrated that pre-incubating PK-15 monolayers with murine IgG2A prior to infection with *M. hyopneumoniae* had no inhibitory effect on adherence (Supplementary Figure 4).

### *M. hyopneumoniae* surface adhesins bind to extracellular actin

To assess the ability of *M. hyopneumoniae* cells to bind to G-actin, we used a microtiter plate binding assay. G-actin bound to the surface of *M. hyopneumoniae* in a dose-dependent manner and reached saturation at 20 μg ml^−1^ (Figure 4A).

**Figure 5:**
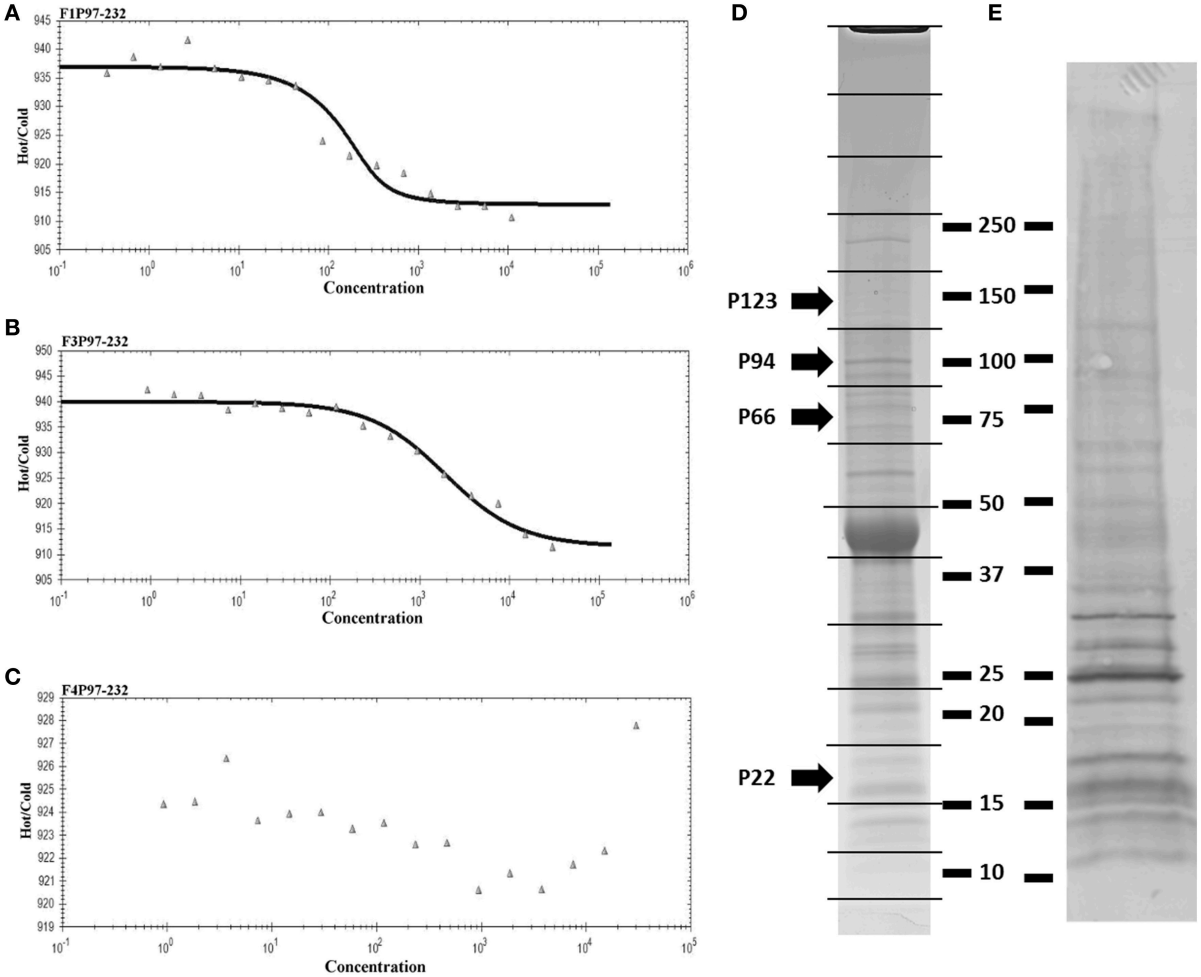
Actin-binding to *M. hyopneumoniae* proteins. Thermophoretic output representing interactions between F1_P97–232_ **(A)**, F3_P97–232_ **(B)**, and F4_P97–232_ **(C)** and actin. Concentration of the non-fluorescence molecule is plotted against the thermophoretic movement of the fluorescent molecule (actin). F1_P97–232_ and F3_P97–232_ bound actin with a *K*_*D*_'s of 34.2 nM and 1.79 μM, respectively. A *K*_*D*_ could not be assigned to F4_P97–232_. **(D)** SDS-PAGE profile of *M. hyopneumoniae* proteins that eluted with trifluoroacetic acid from an avidin column containing biotinylated actin as bait. 143 proteins were identified when each of the 16 slices were digested with trypsin (in gel) and analyzed by LC-MS/MS. P123_J_ (P97 preprotein homolog from strain J) and cleavage fragments of P123_J_ that were identified by LC-MS/MS are indicated by arrows. Molecular weight markers (kDa) are shown on the right. **(E)** Ligand blot depicting *M. hyopneumoniae* whole cell lysate separated by SDS-PAGE and probed with biotinylated (monomeric) G-actin.

As part of a larger investigation to determine the identities of epithelial cell receptors that bind to proteins on the surface of *M. hyopneumoniae*, we coupled avidin agarose with surface-accessible *M. hyopneumoniae* membrane proteins that were labeled with sulfo-NHS-LC-biotin and exposed the derivatized agarose to a native PK-15 cell lysate. After extensive washing, the eluents were digested with trypsin and tryptic peptides were sequenced by LC-MS/MS. Actin, and other proteins with cytoskeletal functions such as vimentin, keratin, tubulin, myosin, and tropomyosin (Supplementary Table 1) comprised 13% of the 105 PK-15 cell-derived proteins that were identified. To further investigate the role of actin as a potential epithelial cell receptor for *M. hyopneumoniae*, we immobilized biotinylated G-actin on avidin agarose beads and incubated them with a whole cell lysate of *M. hyopneumoniae*. After extensive washing to remove unbound proteins, the column was washed with 2M NaCl (13 times physiological concentration) and the remaining bound proteins were eluted using 0.4% trifluoroacetic acid (Figure 5D). 143 *M. hyopneumoniae* proteins were identified by LC-MS/MS (Supplementary Table 2), including most members of the P97 and P102 adhesin families and several cleavage fragments of the archetype cilium adhesin P97. Consistent with data from affinity chromatography experiments, ligand blotting studies showed that biotinylated G-actin bound to many *M. hyopneumoniae* proteins (Figure 5E). Of these 143 proteins, 75 (52%) were found in our surfaceome studies of *M. hyopneumoniae* (Figure 4C and Supplementary Table 2; Berry et al., 2017). On a cautionary note, due to the native conditions under which these experiments were performed, some of the 143 proteins may exist as a complex with *bona fide* actin-binding proteins. As such, these proteins should be considered as putative actin-binding proteins. In order to validate the proteins captured by affinity chromatography as *bona fide* actin-binding proteins, we tested several previously described (Raymond et al., 2015) recombinant fragments of P97 for their ability to bind actin using microscale thermophoresis. The recombinant fragments F1_P97−232_ (amino acids 106–758) and F3_P97−232_ (amino acids 768–938) bound actin with *K*_*D*_'*s* of 34.2 nM and 1.79 μM, respectively (Figures 5A,B). Notably, F4_P97−232_ which contains a multifunctional binding motif with sequence ^1070^K-K-S-S-L-K-V-K-I-T-V-K^1081^ did not bind actin (Figure 5C; Raymond et al., 2015). Taken together, these data indicate that globular β-actin can act as a primary receptor for *M. hyopneumoniae* cells and that a number of surface exposed adhesins are involved in binding to extracellular actin.

## Discussion

LC-MS/MS analysis of affinity-purified, biotinylated surface proteins derived from PK-15 cells identified tryptic peptides that mapped to actin (Supplementary Figure 3). This observation provided the first clue that actin resides on the surface of PK-15 cells. 3D-SIM of non-permeabilized PK-15 cells that were labeled with mAb_β-*act*_ confirmed the extracellular localization of β-actin and, for the first time, *M. hyopneumoniae* interacting intimately with it (Figure 1). Actin localizes on the surface of PK-15 cells in distinct locations, including the cell's leading edge and along actin filaments that stain with phalloidin, a pattern that mirrors the adherence of *M. hyopneumoniae* to PK-15 cells (Figure 1). This is the first observation of *M. hyopneumoniae* colocalizing with extracellular actin and implicates it as a putative bacterial receptor on epithelial cell surfaces.

From this study, there are now several lines of evidence that are consistent with the hypothesis that extracellular actin is an important receptor for *M. hyopneumoniae*: (i) mAb_β-*act*_ can inhibit the ability of *M. hyopneumoniae* to adhere to and colonize PK-15 cells by ~90%; (ii) microtiter plate binding assays show that *M. hyopneumoniae* cells bind to G-actin in a dose dependent and saturable manner; (iii) ligand blotting studies show biotinylated G-actin binds directly to *M. hyopneumoniae* proteins; (iv) microscale thermophoresis showed that recombinant N-terminal F1_P97−232_ and central fragment F3_P97−232_ of the cilium adhesin P97 bind G-actin with *K*_*D*_'*s* of 34.2 nM and 1.79 μM, respectively; (v) affinity chromatography studies using biotinylated *M. hyopneumoniae* surface proteins as bait identified 105 PK-15 cell proteins including actin and other cytoskeletal proteins such as vimentin, keratin, tubulin, myosin, and tropomyosin; (vi) biotinylated G-actin used as bait, recovered 143 *M. hyopneumoniae* proteins including major cilium and extracellular matrix binding proteins. P97 has previously been shown to bind glycosaminoglycans (GAGs), fibronectin, and plasminogen (Minion et al., 2000; Djordjevic et al., 2004; Jenkins et al., 2006; Wilton et al., 2009; Raymond et al., 2015) and now actin. The diversity of molecules presented by epithelial cells that are known to be targeted by adhesins like P97 is consistent with the organism having the capability to adhere to multiple tissue sites and regulate tissue tropism and reinforces the notion that *M. hyopneumoniae* is able to colonize extrapulmonary sites (Lloyd and Etheridge, 1981; Williams and Gallagher, 1981; Yagihashi et al., 1984; Le Carrou et al., 2006; Marois et al., 2007; Woolley et al., 2012). To the best of our knowledge, this is the first study to comprehensively investigate the adherence of a bacterial pathogen to actin; an important cytoskeletal protein that exists in essentially all eukaryote cells.

Porcine epithelial receptors that are targeted by adhesins on the surface of *M. hyopneumoniae* have largely remained uncharacterized. GAGs expressed on the surface of porcine cilia (Erlinger, 1995) have long been known to act as primary receptors for *M. hyopneumoniae* cells (Zhang et al., 1994) and that pre-incubation of *M. hyopneumoniae* cells with cilia or the negatively charged GAGs that decorate cilia can significantly inhibit cilial adherence (Zhang et al., 1994). *M. hyopneumoniae* has also been shown to bind and colocalize with fibronectin at the site of infection (Burnett et al., 2006; Jenkins et al., 2006; Wilton et al., 2009; Deutscher et al., 2010; Seymour et al., 2010, 2011, 2012; Bogema et al., 2011, 2012; Raymond et al., 2013, 2015; Tacchi et al., 2014, 2016; Jarocki et al., 2015) however, little is known about receptors that may be conducive to facilitating chronic infection such as those that are expressed on the surface of denuded epithelium. Coupling biotinylated surface proteins from *M. hyopneumoniae* onto avidin agarose enabled the recovery of PK-15 receptors that were identified using LC-MS/MS. 105 proteins were identified that may be a target of *M. hyopneumoniae* surface proteins, including cytoskeleton-associated proteins such as myosin, keratin, vimentin, and most notably for this work, actin. Actin has been found on the surface of a wide range of eukaryote cells (Chen et al., 1978; Owen et al., 1978; Jones et al., 1979; Bachvaroff et al., 1980; Rubin et al., 1982; Sanders and Craig, 1983; Rosenblatt et al., 1985b; Bach et al., 1986; Pardridge et al., 1989; Por et al., 1991; Moroianu et al., 1993; Dudani and Ganz, 1996; Smalheiser, 1996; Miles et al., 2006; Sandiford et al., 2015; Fu et al., 2017; Sudakov et al., 2017) and is a major constituent of the bronchoalveolar lavage fluid and airway surface liquid that bathes the porcine respiratory epithelium (Bartlett et al., 2013). Collectively, these observations indicate that extracellular actin may be an important receptor for *M. hyopneumoniae*.

Recent surfaceome studies indicate that 159 proteins reside on the surface of *M. hyopneumoniae* (Berry et al., 2017). Of the 143 putative actin-binding proteins identified in this study, 75 were also found on the surface of *M. hyopneumoniae* (Figure 4C) suggesting that a number of these may have roles as adhesins. Furthermore, dominant cleavage fragments of most members of the P97 and P102 adhesin families were recovered from avidin columns coupled with biotinylated actin, including Mhp683, Mhp183, Mhp182, Mhp271, Mhp275, Mhp384, Mhp385, and Mhp493 as well as the cilium adhesin Mhp494 (P159). On a cautionary note, approximately 18% of these 143 proteins possess ATP-binding motifs. Actin monomers contain ATP and it is possible that a proportion of these actin-binding proteins are exploiting an interaction with ATP. Interestingly, many metabolic enzymes and ribosomal proteins were recovered from our affinity chromatography studies and many of these were also detected in surfaceome studies (Berry et al., 2017). These observations suggest that proteins that perform canonical functions in the cytosol have evolved to execute alternate functions on the bacterial cell surface. An excellent example of this is Elongation Factor Tu (Ef-Tu) which has recently been shown to moonlight on the surface of *Staphylococcus aureus, M. hyopneumoniae*, and *Mycoplasma pneumoniae* as a multifunctional adhesin. Notably, rEf-Tu from *M. pneumoniae* bound actin with nanomolar affinity (Widjaja et al., 2017). We also identified Ef-Tu from *M. hyopneumoniae* in our affinity chromatography experiments that used actin as bait (Supplementary Table 2). Phosophoglycerate kinase (PGK) from group B streptococcus (GBS) displays four motifs ^126^KKESK^130^, ^156^HRAH^159^, ^204^KVSDK^208^, ^218^KADK^221^ that play an important role in binding actin and plasminogen (Boone and Tyrrell, 2012). *M. hyopneumoniae* binds plasminogen in a dose-dependent manner and numerous plasminogen binding proteins have been described in this species (Robinson et al., 2013; Jarocki et al., 2015; Raymond and Djordjevic, 2015). In our study, the PGK homolog from *M. hyopneumoniae* was recovered from avidin agarose columns coupled with biotinylated actin (Supplementary Table 2) and it localizes to the cell surface (Berry et al., 2017). Three of the four actin-binding motifs in PGK from GBS are identical in the *M. hyopneumoniae* PGK homolog (data not shown). The motif KKESK has a single amino acid change to KAESK in *M. hyopneumoniae*, however the critical residue (^130^K) for binding actin is conserved (Boone and Tyrrell, 2012). These data suggest that the same motifs in the *M. hyopneumoniae* PGK homolog may facilitate binding to actin and warrants further investigation. In the porcine pathogen *M. suis*, glyceraldehyde 3-phosphate dehydrogenase (GAPDH) (also known as MSP1) binds to actin on the surface of porcine red blood cells (Zhang et al., 2015). When MSG1 was expressed as a recombinant protein in *E. coli* (rMSP1) it trafficked to the cell surface and afforded *E. coli* the ability to adhere to porcine erythrocytes (Zhang et al., 2015) and affinity chromatography experiments using rMSP1 as bait identified β-actin (Zhang et al., 2015). GAPDH from group A streptococci (GAS) interacts with cytoskeletal proteins (Pancholi and Fischetti, 1992) and binds plasminogen and actin in a dose-dependent manner (Seifert et al., 2003). GAPDH is not only expressed on the surface of *M. hyopneumoniae* (Berry et al., 2017) but it was also recovered from our actin affinity chromatography experiments (Supplementary Table 2). Based on these data, surface exposed bacterial moonlighting proteins as well as more classical adhesins, such as P97, have the ability to bind to extracellular actin.

The literature is littered with descriptions of extracellular actin in a diverse array of mammalian cells (Chen et al., 1978; Owen et al., 1978; Jones et al., 1979; Bachvaroff et al., 1980; Rubin et al., 1982; Sanders and Craig, 1983; Rosenblatt et al., 1985b; Bach et al., 1986; Pardridge et al., 1989; Por et al., 1991; Moroianu et al., 1993; Dudani and Ganz, 1996; Smalheiser, 1996; Miles et al., 2006; Sandiford et al., 2015; Fu et al., 2017; Sudakov et al., 2017). Extracellular actin is likely to have evolved to be a multifunctional protein. Consistent with this view, actin is known to act as a so-called danger-associated molecular pattern during cell damage (Ahrens et al., 2012) and modulates neurotransmitter release (Miles et al., 2006). Here we show that *M. hyopneumoniae* binds to globular β-actin and co-localizes with filamentous forms of β-actin. We have characterized a number of putative actin-binding proteins on the surface of *M. hyopneumoniae* and demonstrated that they bind actin.

## Author contributions

BR conceived the confocal experiments and analyzed the 3D-SIM data. He also undertook and analyzed the protein binding and adherence assays. BR also performed the mass spectrometry experiments, analyzed the data and contributed to the drafting of the manuscript. IS prepared samples for confocal microscopy. CU cultured mycoplasma for the initial adherence assays. RM cultured mycoplasma for adherence assays, prepared samples for immunofluorescence microscopy, and aided in image analysis. MR prepared and performed scanning electron microscopy. LT and CW performed the 3D-SIM imaging experiments and assisted with interpretation of the data. MP contributed to the validation of the mass spectrometry data. SD conceived the overall study and drafted the final manuscript.

### Conflict of interest statement

The authors declare that the research was conducted in the absence of any commercial or financial relationships that could be construed as a potential conflict of interest.

## References

[B1] Ahrens S. Zelenay S. Sancho D. Hanc P. Kjaer S. Feest C.. (2012). F-actin is an evolutionarily conserved damage-associated molecular pattern recognized by DNGR-1, a receptor for dead cells. Immunity 36, 635–645. 10.1016/j.immuni.2012.03.008 22483800

[B2] Bach M. A. Lewis D. E. McClure J. E. Parikh N. Rosenblatt H. M. Shearer W. T. (1986). Monoclonal anti-actin antibody recognizes a surface molecule on normal and transformed human B lymphocytes: expression varies with phase of cell cycle. Cell Immunol. 98, 364–374. 10.1016/0008-8749(86)90296-0 3489550

[B3] Bachvaroff R. J. Miller F. Rapaport F. T. (1980). Appearance of cytoskeletal components on the surface of leukemia cells and of lymphocytes transformed by mitogens and Epstein-Barr virus. Proc. Natl. Acad. Sci. U.S.A. 77, 4979–4983. 10.1073/pnas.77.8.4979 6254049 PMC349973

[B4] Bartlett J. A. Albertolle M. E. Wohlford-Lenane C. Pezzulo A. A. Zabner J. Niles R. K.. (2013). Protein composition of bronchoalveolar lavage fluid and airway surface liquid from newborn pigs. Am. J. Physiol. Lung Cell. Mol. Physiol. 305, L256–L66. 10.1152/ajplung.00056.2013 23709621 PMC3743012

[B5] Bereiter M. Young T. F. Joo H. S. Ross R. F. (1990). Evaluation of the ELISA and comparison to the complement fixation test and radial immunodiffusion enzyme assay for detection of antibodies against *Mycoplasma hyopneumoniae* in swine serum. Vet. Microbiol. 25, 177–192. 10.1016/0378-1135(90)90075-7 2126409

[B6] Berry I. J. Jarocki V. M. Tacchi J. L. Raymond B. B. A. Widjaja M. Padula M. P.. (2017). N-terminomics identifies widespread endoproteolysis and novel methionine excision in a genome-reduced bacterial pathogen. Sci. Rep. 7:11063. 10.1038/s41598-017-11296-9 28894154 PMC5593965

[B7] Bogema D. R. Deutscher A. T. Woolley L. K. Seymour L. M. Raymond B. B. Tacchi J. L.. (2012). Characterization of cleavage events in the multifunctional cilium adhesin Mhp684 (P146) reveals a mechanism by which *Mycoplasma hyopneumoniae* regulates surface topography. MBio 3:e00282–11. 10.1128/mBio.00282-11 22493032 PMC3322551

[B8] Bogema D. R. Scott N. E. Padula M. P. Tacchi J. L. Raymond B. B. Jenkins C.. (2011). Sequence TTKF ↓ QE defines the site of proteolytic cleavage in Mhp683 protein, a novel glycosaminoglycan and cilium adhesin of *Mycoplasma hyopneumoniae*. J. Biol. Chem. 286, 41217–41229. 10.1074/jbc.M111.226084 21969369 PMC3308835

[B9] Boone T. J. Tyrrell G. J. (2012). Identification of the actin and plasminogen binding regions of group B streptococcal phosphoglycerate kinase. J. Biol. Chem. 287, 29035–29044. 10.1074/jbc.M112.361261 22761440 PMC3436549

[B10] Bugalhão J. N. Mota L. J. Franco I. S. (2015). Identification of regions within the *Legionella pneumophila* VipA effector protein involved in actin binding and polymerization and in interference with eukaryotic organelle trafficking. Microbiologyopen 5, 118–133. 10.1002/mbo3.316 26626407 PMC4767423

[B11] Burnett T. A. Dinkla K. Rohde M. Chhatwal G. S. Uphoff C. Srivastava M.. (2006). P159 is a proteolytically processed, surface adhesin of *Mycoplasma hyopneumoniae*: defined domains of P159 bind heparin and promote adherence to eukaryote cells. Mol. Microbiol. 60, 669–686. 10.1111/j.1365-2958.2006.05139.x 16629669

[B12] Caruso J. P. Ross R. F. (1990). Effects of *Mycoplasma hyopneumoniae* and Actinobacillus (Haemophilus) pleuropneumoniae infections on alveolar macrophage functions in swine. Am. J. Vet. Res. 51, 227–231. 2301832

[B13] Chen L. B. Murray A. Segal R. A. Bushnell A. Walsh M. L. (1978). Studies on intercellular LETS glycoprotein matrices. Cell 14, 377–391. 10.1016/0092-8674(78)90123-X 667946

[B14] Ciprián A. Pijoan C. Cruz T. Camacho J. Tortora J. Colmenares G.. (1988). *Mycoplasma hyopneumoniae* increases the susceptibility of pigs to experimental *Pasteurella multocida* pneumonia. Can. J. Vet. Res. 52, 434–438. 3196973 PMC1255487

[B15] Clark L. K. Armstrong C. H. Scheidt A. B. Van Alistine W. G. (1993). The effect of *Mycoplasma hyopneumoniae* infection on growth in pigs with or without environmental constraints. Swine Health Prod. 1, 10–14.

[B16] DeBey M. C. Ross R. F. (1994). Ciliostasis and loss of cilia induced by *Mycoplasma hyopneumoniae* in porcine tracheal organ cultures. Infect. Immun. 62, 5312–5318. 7960110 10.1128/iai.62.12.5312-5318.1994 PMC303270

[B17] Deutscher A. T. Jenkins C. Minion F. C. Seymour L. M. Padula M. P. Dixon N. E.. (2010). Repeat regions R1 and R2 in the P97 paralogue Mhp271 of *Mycoplasma hyopneumoniae* bind heparin, fibronectin and porcine cilia. Mol. Microbiol. 78, 444–458. 10.1111/j.1365-2958.2010.07345.x 20879998

[B18] Djordjevic S. P. Cordwell S. J. Djordjevic M. A. Wilton J. Minion F. C. (2004). Proteolytic processing of the *Mycoplasma hyopneumoniae* cilium adhesin. Infect. Immun. 72, 2791–2802. 10.1128/IAI.72.5.2791-2802.2004 15102789 PMC387856

[B19] Dudani A. K. Ganz P. R. (1996). Endothelial cell surface actin serves as a binding site for plasminogen, tissue plasminogen activator and lipoprotein(a). Br. J. Haematol. 95, 168–178. 10.1046/j.1365-2141.1996.7482367.x 8857956

[B20] Erlinger R. (1995). Glycosaminoglycans in porcine lung: an ultrastructural study using cupromeronic blue. Cell Tissue Res. 281, 473–483. 10.1007/BF00417864 7553767

[B21] Fu L. Han L. Xie C. Li W. Lin L. Pan S.. (2017). Identification of extracellular actin as a ligand for triggering receptor expressed on myeloid cells-1 signaling. Front. Immunol. 8:917. 10.3389/fimmu.2017.00917 28824642 PMC5545922

[B22] Goodwin R. F. W. Pomeroy A. P. Whittles P. (1967). Characterization of Mycoplasma suipneumoniae - a mycoplasma causing enzootic pneumonia of pigs. J. Hyg. 65, 85–96. 10.1017/S0022172400045563 4960045 PMC2130190

[B23] Herman I. M. (1993). Actin isoforms. Curr. Opin. Cell Biol. 5, 48–55. 10.1016/S0955-0674(05)80007-9 8448030

[B24] Holst S. Yeske P. Pieters M. (2015). Elimination of *Mycoplasma hyopneumoniae* from breed-to-wean farms: a review of current protocols with emphasis on herd closure and medication. J. Swine Health Prod. 23, 321–330.

[B25] Jarocki V. M. Santos J. Tacchi J. L. Raymond B. B. Deutscher A. T. Jenkins C.. (2015). MHJ_0461 is a multifunctional leucine aminopeptidase on the surface of *Mycoplasma hyopneumoniae*. Open Biol. 5:140175. 10.1098/rsob.140175 25589579 PMC4313372

[B26] Jenkins C. Wilton J. L. Minion F. C. Falconer L. Walker M. J. Djordjevic S. P. (2006). Two domains within the *Mycoplasma hyopneumoniae* cilium adhesin bind heparin. Infect. Immun. 74:7. 10.1128/IAI.74.1.481-487.2006 16369004 PMC1346629

[B27] Jones P. A. Scott-Burden T. Gevers W. (1979). Glycoprotein, elastin, and collagen secretion by rat smooth muscle cells. Proc. Natl. Acad. Sci. U.S.A. 76, 353–357. 10.1073/pnas.76.1.353 284351 PMC382937

[B28] Kobisch M. Friis N. F. (1996). Swine mycoplasmoses. Rev. Sci. Tech. 15, 1569–1605. 10.20506/rst.15.4.983 9190026

[B29] Le Carrou J. Laurentie M. Kobisch M. Gautier-Bouchardon A. V. (2006). Persistence of *Mycoplasma hyopneumoniae* in experimentally infected pigs after marbofloxacin treatment and detection of mutations in the parC gene. Antimicrob. Agents Chemother. 50, 1959–1966. 10.1128/AAC.01527-05 16723552 PMC1479153

[B30] Lloyd L. C. Etheridge J. R. (1981). The pathological and serological response induced in pigs by parenteral inoculation of *Mycoplasma hyopneumoniae*. J. Comp. Pathol. 91, 77–83. 10.1016/0021-9975(81)90047-5 7343577

[B31] Maes D. Segales J. Meyns T. Sibila M. Pieters M. Haesebrouck F. (2008). Control of *Mycoplasma hyopneumoniae* infections in pigs. Vet. Microbiol. 126, 297–309. 10.1016/j.vetmic.2007.09.008 17964089 PMC7130725

[B32] Mare C. J. Switzer W. P. (1965). New species: *Mycoplasma Hyopneumoniae*; a causative agent of virus pig pneumonia. Vet. Med. Small Anim. Clin. 60, 841–846. 14323369

[B33] Marois C. Le Carrou J. Kobisch M. Gautier-Bouchardon A. V. (2007). Isolation of *Mycoplasma hyopneumoniae* from different sampling sites in experimentally infected and contact SPF piglets. Vet. Microbiol. 120, 96–104. 10.1016/j.vetmic.2006.10.015 17116374

[B34] Miles L. A. Andronicos N. M. Baik N. Parmer R. J. (2006). Cell-surface actin binds plasminogen and modulates neurotransmitter release from catecholaminergic cells. J. Neurosci. 26, 13017–13024. 10.1523/JNEUROSCI.2070-06.2006 17167091 PMC6674961

[B35] Minion F. C. Adams C. Hsu T. (2000). R1 region of P97 mediates adherence of *Mycoplasma hyopneumoniae* to swine cilia. Infect. Immun. 68, 3056–3060. 10.1128/IAI.68.5.3056-3060.2000 10769015 PMC97530

[B36] Moroianu J. Fett J. W. Riordan J. F. Vallee B. L. (1993). Actin is a surface component of calf pulmonary artery endothelial cells in culture. Proc. Natl. Acad. Sci. U.S.A. 90, 3815–3819. 10.1073/pnas.90.9.3815 8483899 PMC46396

[B37] Opriessnig T. Thacker E. L. Yu S. Fenaux M. Meng X. J. Halbur P. G. (2004). Experimental reproduction of postweaning multisystemic wasting syndrome in pigs by dual infection with *Mycoplasma hyopneumoniae* and porcine circovirus type 2. Vet. Pathol. 41, 624–640. 10.1354/vp.41-6-624 15557072

[B38] Otterbein L. R. Graceffa P. Dominguez R. (2001). The crystal structure of uncomplexed actin in the ADP state. Science 293, 708–711. 10.1126/science.1059700 11474115

[B39] Owen M. J. Auger J. Barber B. H. Edwards A. J. Walsh F. S. Crumpton M. J. (1978). Actin may be present on the lymphocyte surface. Proc. Natl. Acad. Sci. U.S.A. 75, 4484–4488. 10.1073/pnas.75.9.4484 309133 PMC336140

[B40] Pallarés F. J. Halbur P. G. Opriessnig T. Sorden S. D. Villar D. Janke B. H.. (2002). Porcine circovirus type 2 (PCV-2) coinfections in US field cases of postweaning multisystemic wasting syndrome (PMWS). J. Vet. Diagn. Invest. 14, 515–519. 10.1177/104063870201400614 12423038

[B41] Pancholi V. Fischetti V. A. (1992). A major surface protein on group A streptococci is a glyceraldehyde-3-phosphate-dehydrogenase with multiple binding activity. J. Exp. Med. 176, 415–426. 10.1084/jem.176.2.415 1500854 PMC2119316

[B42] Pardridge W. M. Nowlin D. M. Choi T. B. Yang J. Calaycay J. Shively J. E. (1989). Brain capillary 46,000 dalton protein is cytoplasmic actin and is localized to endothelial plasma membrane. J. Cereb. Blood Flow Metab. 9, 675–680. 10.1038/jcbfm.1989.95 2777936

[B43] Pendarvis K. Padula M. P. Tacchi J. L. Petersen A. C. Djordjevic S. P. Burgess S. C.. (2014). Proteogenomic mapping of *Mycoplasma hyopneumoniae* virulent strain 232. BMC Genomics 15:576. 10.1186/1471-2164-15-576 25005615 PMC4102725

[B44] Por S. B. Cooley M. A. Breit S. N. Penny R. French P. W. (1991). Antibodies to tubulin and actin bind to the surface of a human monocytic cell line, U937. J. Histochem. Cytochem. 39, 981–985. 10.1177/39.7.1865114 1865114

[B45] Raymond B. B. Djordjevic S. (2015). Exploitation of plasmin(ogen) by bacterial pathogens of veterinary significance. Vet. Microbiol. 178, 1–13. 10.1016/j.vetmic.2015.04.008 25937317

[B46] Raymond B. B. Jenkins C. Seymour L. M. Tacchi J. L. Widjaja M. Jarocki V. M.. (2015). Proteolytic processing of the cilium adhesin MHJ_0194 (P123J) in *Mycoplasma hyopneumoniae* generates a functionally diverse array of cleavage fragments that bind multiple host molecules. Cell. Microbiol. 17, 425–444. 10.1111/cmi.12377 25293691

[B47] Raymond B. B. Tacchi J. L. Jarocki V. M. Minion F. C. Padula M. P. Djordjevic S. P. (2013). P159 from *Mycoplasma hyopneumoniae* binds porcine cilia and heparin and is cleaved in a manner akin to ectodomain shedding. J. Proteome Res. 12, 5891–5903. 10.1021/pr400903s 24195521

[B48] Robinson M. W. Buchtmann K. A. Jenkins C. Tacchi J. L. Raymond B. B. To J.. (2013). MHJ_0125 is an M42 glutamyl aminopeptidase that moonlights as a multifunctional adhesin on the surface of *Mycoplasma hyopneumoniae*. Open Biol. 3:130017. 10.1098/rsob.130017 23594879 PMC3718333

[B49] Rosenblatt H. M. Parikh N. McClure J. E. Meza I. Hwo S. Y. Bryan J.. (1985a). Mitogen-like monoclonal anti-actin antibodies. J. Immunol. 135, 995–1000. 4008931

[B50] Rosenblatt H. M. Ulrich R. G. Shearer W. T. (1985b). Monoclonal anti-actin antibody modulates expression of surface antigen on L cells. Res. Commun. Chem. Pathol. Pharmacol. 49, 35–45. 3929344

[B51] Rubin R. W. Suchard S. J. Ozores J. N. (1982). Less membrane-associated actin in transformed versus normal rat kidney cells. Cell Biol. Int. Rep. 6, 177–187. 10.1016/0309-1651(82)90095-9 7199391

[B52] Sanders S. K. Craig S. W. (1983). A lymphocyte cell surface molecule that is antigenically related to actin. J. Immunol. 131, 370–377. 6190917

[B53] Sandiford S. L. Dong Y. Pike A. Blumberg B. J. Bahia A. C. Dimopoulos G. (2015). Cytoplasmic actin is an extracellular insect immune factor which is secreted upon immune challenge and mediates phagocytosis and direct killing of bacteria, and is a Plasmodium Antagonist. PLoS Pathog. 11:e1004631. 10.1371/journal.ppat.1004631 25658622 PMC4450071

[B54] Scarman A. L. Chin J. C. Eamens G. J. Delaney S. F. Djordjevic S. P. (1997). Identification of novel species-specific antigens of *Mycoplasma hyopneumoniae* by preparative SDS-PAGE ELISA profiling. Microbiology 143(Pt 2), 663–673. 10.1099/00221287-143-2-663 9043142

[B55] Seifert K. N. McArthur W. P. Bleiweis A. S. Brady L. J. (2003). Characterization of group B streptococcal glyceraldehyde-3-phosphate dehydrogenase: surface localization, enzymatic activity, and protein-protein interactions. Can. J. Microbiol. 49, 350–356. 10.1139/w03-042 12897829

[B56] Seymour L. M. Deutscher A. T. Jenkins C. Kuit T. A. Falconer L. Minion F. C.. (2010). A processed multidomain *Mycoplasma hyopneumoniae* adhesin binds fibronectin, plasminogen, and swine respiratory cilia. J. Biol. Chem. 285, 33971–33978. 10.1074/jbc.M110.104463 20813843 PMC2962497

[B57] Seymour L. M. Falconer L. Deutscher A. T. Minion F. C. Padula M. P. Dixon N. E.. (2011). Mhp107 is a member of the multifunctional adhesin family of *Mycoplasma hyopneumoniae*. J. Biol. Chem. 286, 10097–10104. 10.1074/jbc.M110.208140 21245147 PMC3060461

[B58] Seymour L. M. Jenkins C. Deutscher A. T. Raymond B. B. Padula M. P. Tacchi J. L.. (2012). Mhp182 (P102) binds fibronectin and contributes to the recruitment of plasmin(ogen) to the *Mycoplasma hyopneumoniae* cell surface. Cell. Microbiol. 14, 81–94. 10.1111/j.1462-5822.2011.01702.x 21951786

[B59] Smalheiser N. R. (1996). Proteins in unexpected locations. Mol. Biol. Cell 7, 1003–1014. 10.1091/mbc.7.7.1003 8862516 PMC275954

[B60] Stipkovits L. Miller D. Glavits R. Fodor L. Burch D. (2001). Treatment of pigs experimentally infected with *Mycoplasma hyopneumoniae, Pasteurella multocida*, and Actinobacillus pleuropneumoniae with various antibiotics. Can. J. Vet. Res. 65, 213–222. 11768127 PMC1189682

[B61] Strauss M. P. Liew A. T. Turnbull L. Whitchurch C. B. Monahan L. G. Harry E. J. (2012). 3D-SIM super resolution microscopy reveals a bead-like arrangement for FtsZ and the division machinery: implications for triggering cytokinesis. PLoS Biol. 10:e1001389. 10.1371/journal.pbio.1001389 22984350 PMC3439403

[B62] Sudakov N. P. Klimenkov I. V. Byvaltsev V. A. Nikiforov S. B. Konstantinov Y. M. (2017). Extracellular actin in health and disease. Biochem. Mosc. 82, 1–12. 10.1134/S0006297917010011 28320282

[B63] Tacchi J. L. Raymond B. B. Haynes P. A. Berry I. J. Widjaja M. Bogema D. R.. (2016). Post-translational processing targets functionally diverse proteins in *Mycoplasma hyopneumoniae*. Open Biol. 6:150210. 10.1098/rsob.150210 26865024 PMC4772806

[B64] Tacchi J. L. Raymond B. B. Jarocki V. M. Berry I. J. Padula M. P. Djordjevic S. P. (2014). Cilium adhesin P216 (MHJ_0493) is a target of ectodomain shedding and aminopeptidase activity on the surface of *Mycoplasma hyopneumoniae*. J. Proteome Res. 13, 2920–2930. 10.1021/pr500087c 24804907

[B65] Thacker E. L. Thacker B. J. Janke B. H. (2001). Interaction between *Mycoplasma hyopneumoniae* and swine influenza virus. J. Clin. Microbiol. 39, 2525–2530. 10.1128/JCM.39.7.2525-2530.2001 11427564 PMC88180

[B66] Thacker E. L. Thacker B. J. Young T. F. Halbur P. G. (2000). Effect of vaccination on the potentiation of porcine reproductive and respiratory syndrome virus (PRRSV)-induced pneumonia by *Mycoplasma hyopneumoniae*. Vaccine 18, 1244–1252. 10.1016/S0264-410X(99)00395-3 10649626

[B67] Widjaja M. Harvey K. L. Hagemann L. Berry I. J. Jarocki V. M. Raymond B. B. A.. (2017). Elongation factor Tu is a multifunctional and processed moonlighting protein. Sci. Rep. 7:11227. 10.1038/s41598-017-10644-z 28894125 PMC5593925

[B68] Williams P. P. Gallagher J. E. (1981). Effects of *Mycoplasma hyopneumoniae* and *M. hyorhinis* on ependymal cells of the porcine lateral ventricles as observed by scanning and transmission electron microscopy. Scan. Electron Microsc. 4, 133–140. 7347417

[B69] Wilton J. Jenkins C. Cordwell S. J. Falconer L. Minion F. C. Oneal D. C.. (2009). Mhp493 (P216) is a proteolytically processed, cilium and heparin binding protein of *Mycoplasma hyopneumoniae*. Mol. Microbiol. 71, 566–582. 10.1111/j.1365-2958.2008.06546.x 19040640

[B70] Woolley L. K. Fell S. Gonsalves J. R. Walker M. J. Djordjevic S. P. Jenkins C.. (2012). Evaluation of clinical, histological and immunological changes and qPCR detection of *Mycoplasma hyopneumoniae* in tissues during the early stages of mycoplasmal pneumonia in pigs after experimental challenge with two field isolates. Vet. Microbiol. 161, 186–195. 10.1016/j.vetmic.2012.07.025 22863144

[B71] Wyrsch E. Roy Chowdhury P. Abraham S. Santos J. Darling A. E. Charles I. G.. (2015). Comparative genomic analysis of a multiple antimicrobial resistant enterotoxigenic *E. coli* O157 lineage from Australian pigs. BMC Genomics 16:165. 10.1186/s12864-015-1382-y 25888127 PMC4384309

[B72] Yagihashi T. Nunoya T. Mitui T. Tajima M. (1984). Effect of *Mycoplasma hyopneumoniae* infection on the development of Haemophilus pleuropneumoniae pneumonia in pigs. Nippon. Juigaku Zasshi 46, 705–713. 10.1292/jvms1939.46.705 6513243

[B73] Zhang Q. Young T. F. Ross R. F. (1994). Microtiter plate adherence assay and receptor analogs for *Mycoplasma hyopneumoniae*. Infect. Immun. 62, 1616–1622. 8168922 10.1128/iai.62.5.1616-1622.1994 PMC186367

[B74] Zhang Y. Zou Y. Ma P. Muhammad H. M. Li Y. Jiang P. (2015). Identification of *Mycoplasma suis* MSG1 interaction proteins on porcine erythrocytes. Arch. Microbiol. 197, 277–283. 10.1007/s00203-014-1050-7 25344885

[B75] Zhu W. Zhang X. Yang J. Xu W. Xu M. (2015). [Simultaneous determination of multi-classes of veterinary drug residues in pork by ultra performance liquid chromatography coupled with quadrupole-time of flight mass spectrometry]. Se Pu 33, 1002–1008. 10.3724/SP.J.1123.2015.04033 26753290

[B76] Zielinski G. C. Young T. Ross R. F. Rosenbusch R. F. (1990). Adherence of *Mycoplasma hyopneumoniae* to cell monolayers. Am. J. Vet. Res. 51, 339–343. 2316909

