# Supplementary material for: Extracellular Actin Is a Receptor for Mycoplasma hyopneumoniae
Source: Front Cell Infect Microbiol. 2018 Feb 27;8:54. doi: 10.3389/fcimb.2018.00054 (PMC5835332; doi:10.3389/fcimb.2018.00054)
Supplement: Supplementary file 1 [file DataSheet1.docx]

Supplementary Material

Extracellular actin is a receptor for *Mycoplasma hyopneumoniae*

Raymond BBA^1^, Madhkoor R^1^, Schleicher I^2^, Uphoff CC^3^, Turnbull L^1^, Whitchurch CB^1^, Rohde M^2^, Padula MP^1,4^, and Djordjevic SP^1,4*^

* Corresponding author: Steven. P. Djordjevic


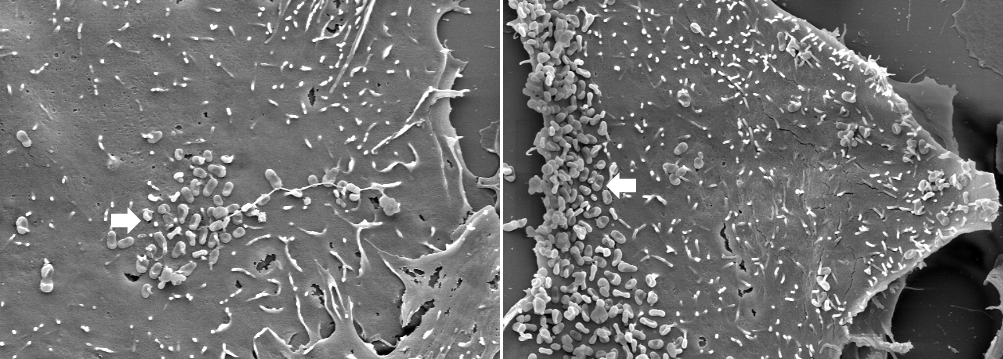


Supplementary Figure 1: Low resolution (5000 × magnification) SEM image of *M. hyopneumoniae* cells adhering to PK-15 cells. The arrow in the left panel depicts a discrete area on the surface of a PK-15 cell that is colonised by *M. hyopneumoniae* cells. The arrow in the right panel depicts *M. hyopneumoniae* cells preferentially adhering to the leading edge of the PK-15 monolayer.


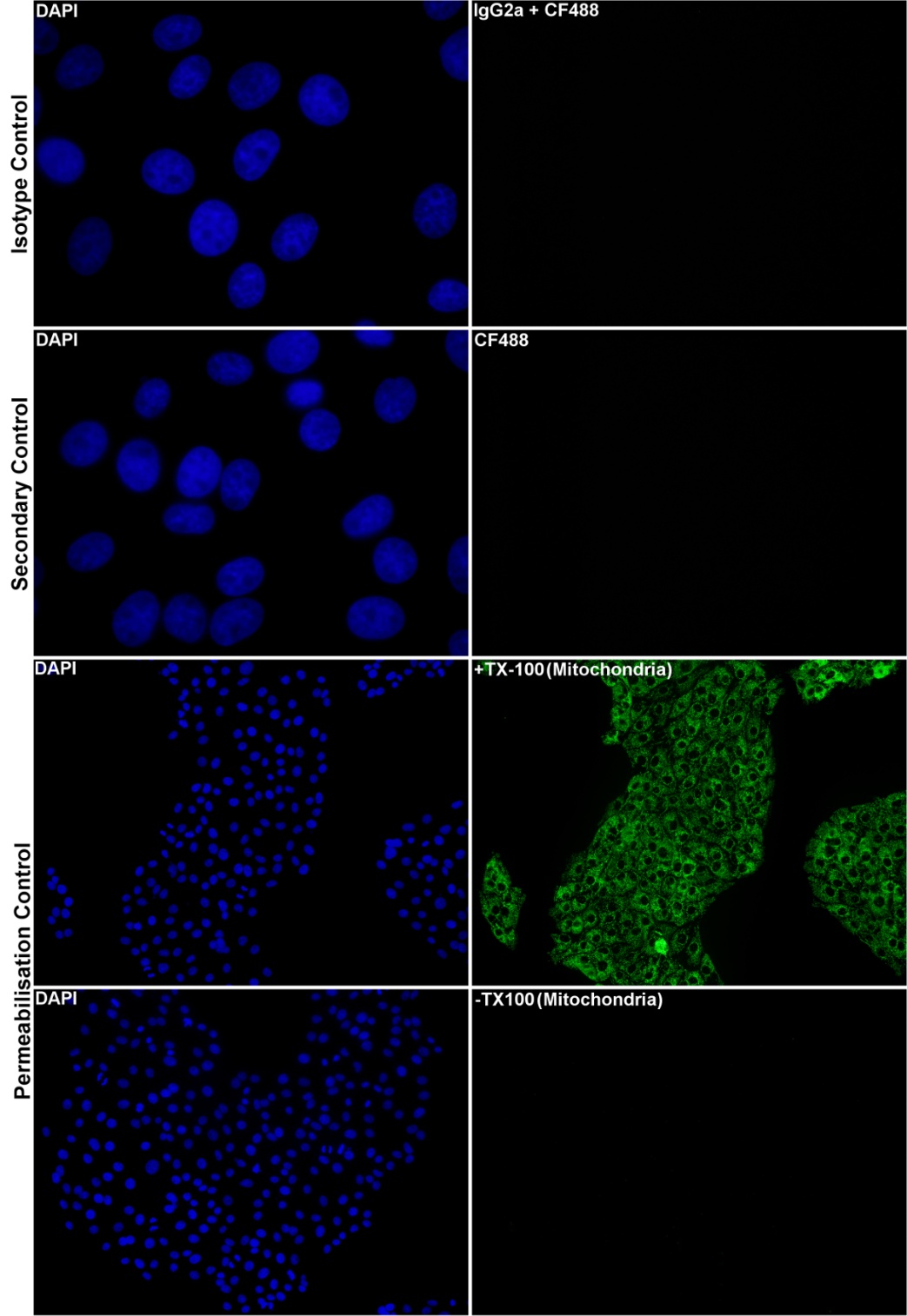


Supplementary Figure 2: Antibody control experiments in uninfected PK-15 cells. For isotype controls, PK-15 cells were incubated with murine IgG2a, followed by incubation with anti-murine CF™ 488-conjugated secondary antibodies. For secondary antibody controls, PK-15 cells were incubated with anti-murine CF™ 488-conjugated secondary antibodies. PK-15 cells were then stained with DAPI post-permeabilization. As can be seen, there is no non-specific binding of either antibody to PK-15 cells. For permeabilization controls, paraformaldehyde-fixed PK-15 cells were either permeabilised in Triton X-100 or left unpermeabilised. Murine monoclonal antibodies against mitochondria were incubated with the samples overnight, followed by incubation with anti-murine CF™ 488-conjugated secondary antibodies. As can be seen in the cells that were permeabilised with Triton X-100, there is intense staining of the mitochondria, whereas cells that remained unpermeabilised exhibited no such staining. All images are representative of 10 fields of view captured for each sample.


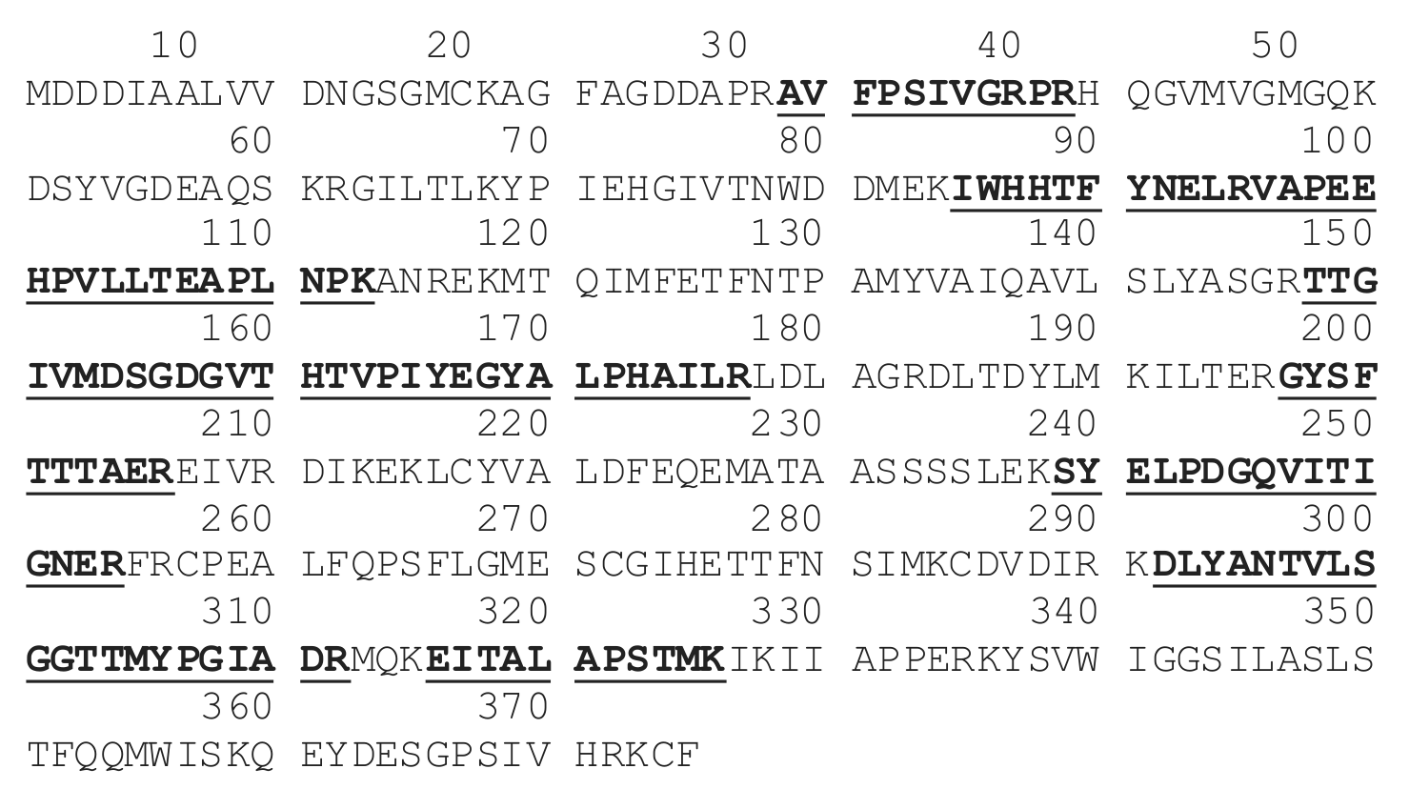


Supplementary Figure 3: Peptide coverage of biotinylated actin (Q6QAQ1) recovered from the surface of PK-15 epithelial-like cells (*Sus scrofa*). Biotinylated PK-15 surface proteins were incubated with avidin agarose and purified by elution using low pH. Bound proteins were separated by SDS PAGE and analysed by LC-MS/MS. The peptide coverage presented here was obtained from a region of the gel at approximately 40 kDa.


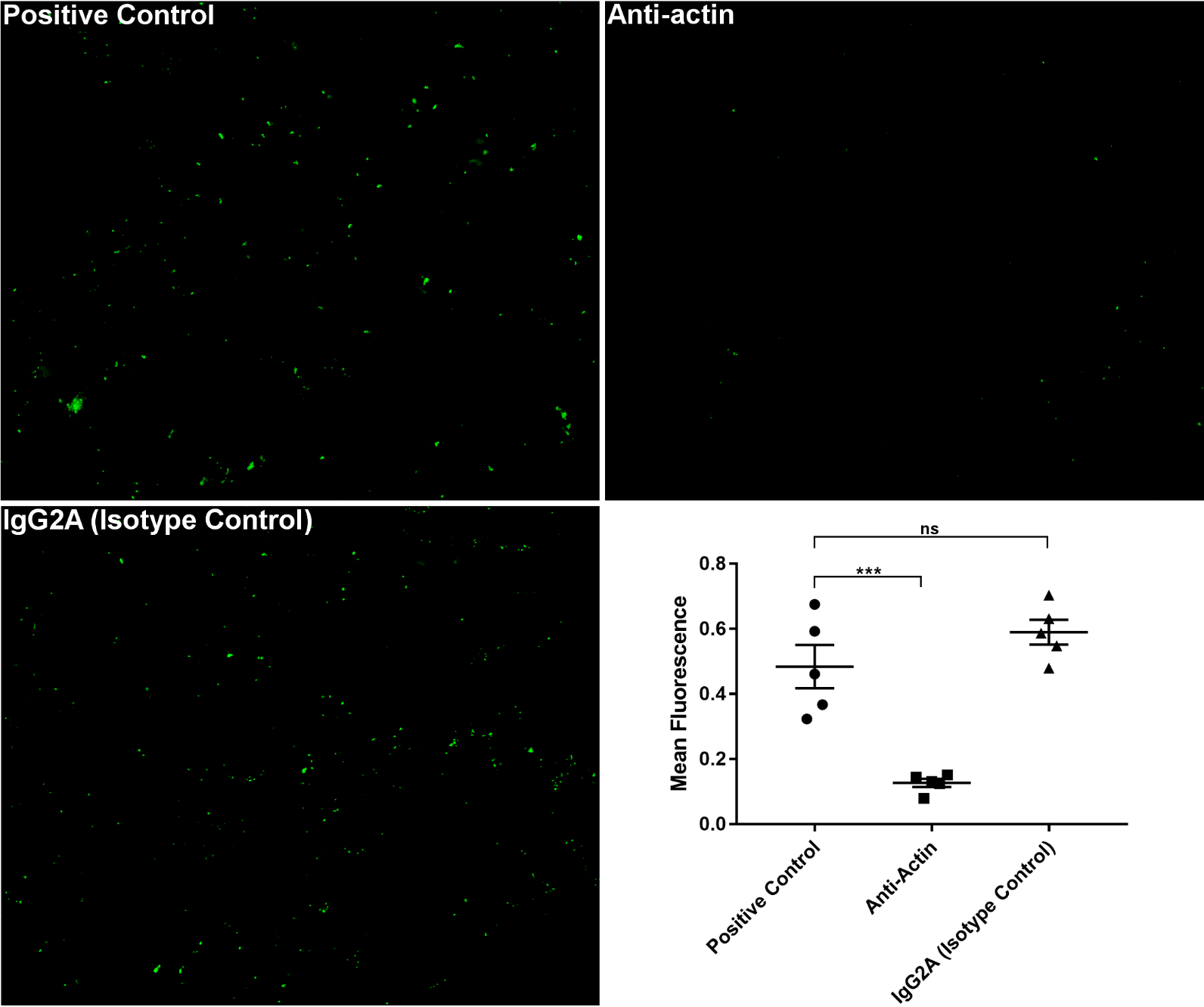


Supplementary Figure 4: Isotype controls in PK-15 cells infected with *M. hyopneumoniae*. PK-15 monolayers were pre-incubated with either mAb_β-act_ (Anti-actin), murine IgG2A (Isotype Control), or no antibody (Positive Control) for 2 h prior to infection with *M. hyopneumoniae*. *M. hyopneumoniae* cells were labelled with F2_P94-J_ antisera conjugated to CF™ 488 (green). Five fields of views were imaged at 20 × magnification, corresponding to adherence across approximately 7, 500 PK-15 cells per sample type. Fluorescence intensity correlating to adherent *M. hyopneumoniae* cells was compared across samples using an unpaired t-test. There was a statistical difference between the positive control and cells pre-incubated with mAb_β-act_ (P-value = 0.0007) and no statistical difference between the positive control and isotype control (P-value = 0.2046). These results reiterate that mAb_β-act_ competes with *M. hyopneumoniae* for binding sites on the PK-15 cell surface, and that this is not attributed to non-specific binding by IgG2A.


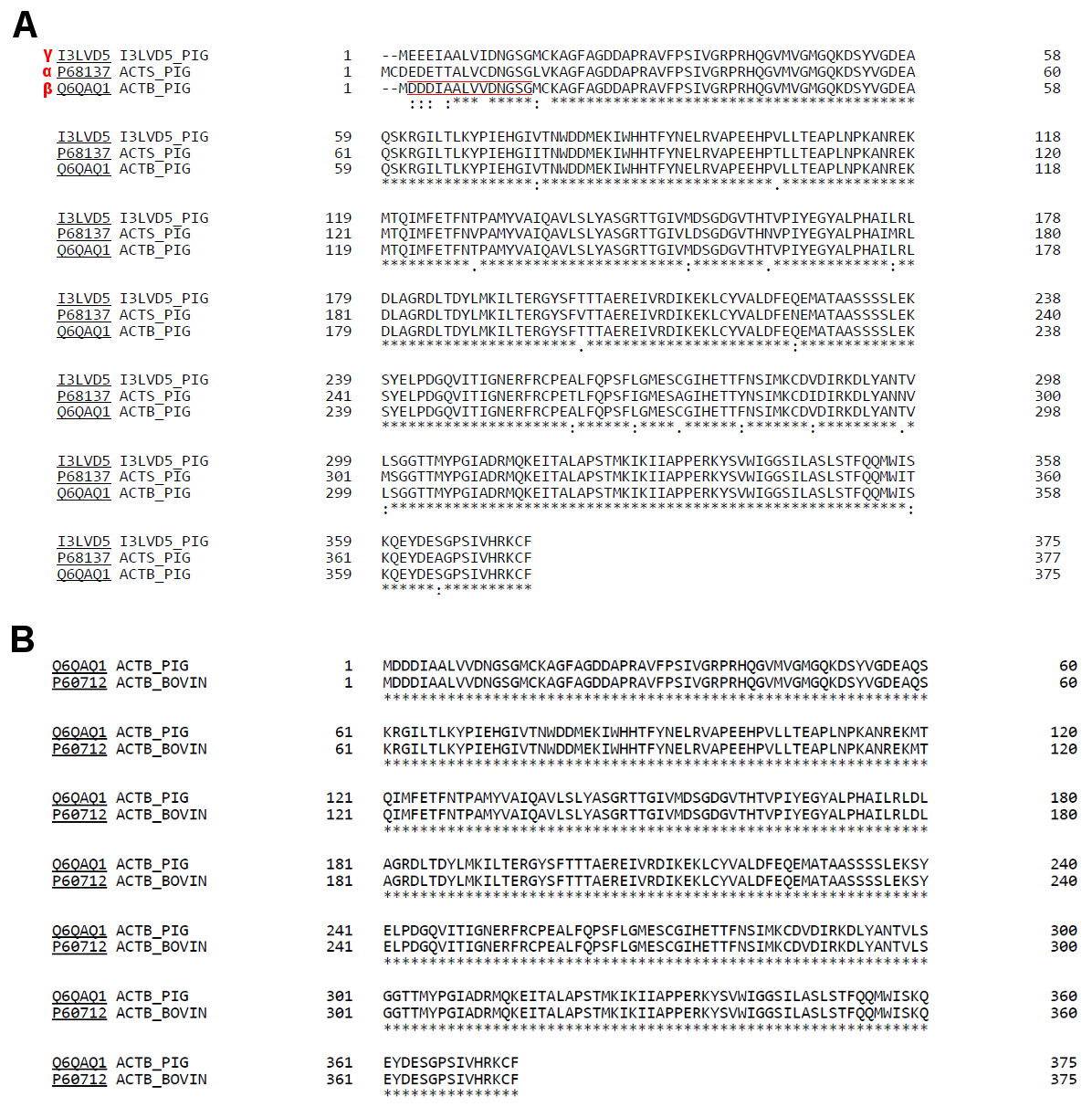


Supplementary Figure 5: Sequence alignments of actin isoforms performed using UniProt. A) Based on alignments, α-, β- and γ- actin (*Sus scrofa*) share 92.5% sequence identity, only differing significantly in the N-terminal 20 amino acids. The red lines indicate the sequence that mAb_β-act_ recognises. B) Sequence alignments of β-actin from *Bos taurus* and *Sus scrofa* demonstrating 100% sequence identity. Asterisks indicate amino acid residues that are identical between sequences, double dots indicate strong similarity, single dots indicate weak similarity and no mark indicates a dissimilar amino acid substitution.

Supplementary Table 1: Porcine proteins purified by the biotinylated *M. hyopneumoniae* affinity column.

| **Accession** | **Protein Name** | **Elution** |
| --- | --- | --- |
| ***Cytoskeletal proteins*** | | |
| F1S0J8 | Keratin19 | TFA |
| F1S6M7 | Tubulin beta | Salt |
| F1SGG2 | Keratin8 | TFA |
| F1SHC1 | Tubulin alpha | Salt + TFA |
| F1SKJ1 | Myosin 9 | Salt |
| F1SM78 | Myosin regulatory light chain | Salt + TFA |
| F2Z571 | Tubulin beta 2C chain | TFA |
| F2Z5B6 | Tropomyosin alpha-1 chain | Salt |
| K7GK75 | Cofilin 1 | Salt + TFA |
| P02543 | Vimentin | TFA |
| P60662 | Myosin light polypeptide 6 | Salt |
| P60662-2 | Isoform Smooth muscle of Myosin light polypeptide 6 | TFA |
| Q6QAQ1 | Actin, cytoplasmic 1 | Salt + TFA |
| Q8WNW3 | Junction plakoglobin | Salt |
| ***Ribosomal proteins*** | | |
| B7TJ03 | Ribosomal protein L26-like 1 | Salt |
| F1RQ91 | 40S ribosomal protein S4, Y isoform 1 | TFA |
| F1RTJ9 | 60S ribosomal protein L21 | TFA |
| F1RYZ0 | 60S acidic ribosomal protein P2 | TFA |
| F1RZ28 | 40S ribosomal protein S10 | Salt + TFA |
| F1RGG1 | 40S ribosomal protein S19 | Salt + TFA |
| F1S4M2 | 40S ribosomal protein S18 | TFA |
| F1SJQ6 | 60S ribosomal protein L14 | TFA |
| F1SNY2 | 60S ribosomal protein L3 | Salt + TFA |
| F2Z522 | Ribosomal protein L23a | Salt |
| F2Z539 | 40S ribosomal protein S20 | Salt + TFA |
| F2Z546 | 60S ribosomal protein L19 | TFA |
| F2Z554 | Ribosomal protein L30 | TFA |
| F2Z568 | Ribosomal protein L38 | TFA |
| F2Z5C7 | 40S ribosomal protein S3a-A | TFA |
| F2Z5E6 | 40S ribosomal protein S5 | TFA |
| F2Z5F5 | 40S ribosomal protein S8 | TFA |
| F2Z5G8 | 40S ribosomal protein S25 | TFA |
| F2Z5Q2 | 60S ribosomal protein L24 | TFA |
| F2Z5Q6 | 40S ribosomal protein S6 | TFA |
| F6Q5P0 | 40S ribosomal protein S13 | TFA |
| I3L5B2 | 40S ribosomal protein S7 | Salt + TFA |
| I3L6F1 | 60S ribosomal protein L18 | TFA |
| I3LD74 | 60S ribosomal protein L11 | Salt |
| I3LJP6 | 40S ribosomal protein S24 | TFA |
| I3LSD3 | 60S ribosomal protein L13 | TFA |
| P62831 | 60S ribosomal protein L23 | TFA |
| P62863 | 40S ribosomal protein S30 | Salt |
| P62901 | 60S ribosomal protein L31 | Salt |
| P63053 | Ubiquitin-60S ribosomal protein L40 | Salt |
| P67985 | 60S ribosomal protein L22 | Salt + TFA |
| Q0Z8U2 | 40S ribosomal protein S3 | TFA |
| Q29201 | 40S ribosomal protein S16 | TFA |
| Q6QAS5 | 60S ribosomal protein L12 | TFA |
| ***Translational & chaperone proteins*** | | |
| B9W5V0 | Elongation factor 1-alpha | Salt + TFA |
| F1RFI1 | Elongation factor Tu | TFA |
| F1RJS2 | Clusterin | TFA |
| F1RY92 | Serine/arginine-rich splicing factor 3 | Salt |
|  |  |  |
| F1SMZ7 | Mitochondrial heat shock 60 kDa protein 1 | TFA |
| F1S415 | BAG family molecular chaperone regulator 3 | TFA |
|  |  |  |
| F1SQ46 | Cold shock domain protein A (DNA binding) | Salt |
| I3LLG4 | DnaJ | TFA |
| ***Serum proteins*** | | |
| B3CL06 | Serotransferrin | TFA |
| F1RPW2 | Coagulation factor V light chain | Salt |
| F1RUN2 | Serum albumin | TFA |
| F1RUQ0 | Immunoglobulin J | Salt + TFA |
| I3LFH1 | IgA heavy chian constant region | TFA |
| K7GNC6 | Apolipoprotein O-like protein | TFA |
| L8B0W9 | IgG heavy chain | Salt + TFA |
| P18648 | Apolipoprotein A-I | TFA |
| ***Nuclear associated proteins*** | | |
| B6CVL5 | High mobility group AT-hook 1 transcript variant 2 | Salt |
| D5KJI2 | High mobility group AT-hook protein 1 | Salt |
| F1RFQ7 | GTP-binding nuclear protein Ran | TFA |
| ***Non-Grouped & putative uncharacterised proteins*** | | |
| B2ZF46 | ATP synthase subunit alpha | TFA |
| ENSSSCP00000018094 | GTPase-activating protein-binding protein | Salt |
| F1S827 | Isoform 3 of Plasminogen activator inhibitor 1 RNA-binding protein | TFA |
| F1S8L9 | Heterogeneous nuclear ribonucleoprotein U | TFA |
| F1SA40 | V-type proton ATPase subunit D | TFA |
| F1SAK6 | ATP synthase subunit g, mitochondrial | TFA |
| F1SEY8 | Polymeric immunoglobulin receptor | TFA |
| F1SFF4 | Chromosome 14 open reading frame 166 | TFA |
| F1SFI5 | Histidine-rich glycoprotein | TFA |
| F1SLR1 | NADH Dehydrogenase | Salt + TFA |
| F1SM14 | Secreted phosphoprotein 24 | TFA |
| F1SPG1 | Protein H1fx | Salt |
| F1SRQ7 | Single-stranded DNA-binding protein | Salt |
| F2Z578 | Histone H2B | TFA |
| F2Z5G3 | Calmodulin 2 | Salt |
| G8ENL4 | FUS RNA binding protein | Salt |
| I3LI24 | Guanine nucleotide-binding protein subunit beta-2-like 1 | TFA |
| I3LNZ2 | Histone H1.0 | Salt |
| I3LPW0 | NADH Dehydrogenase | TFA |
| I3LRH2 | Nucleolin-related protein | Salt |
| I3LS16 | NADH dehydrogenase [ubiquinone] 1 alpha subcomplex subunit 2 | Salt |
| I3LS73 | Mitochondrial import inner membrane translocase subunit Tim13 | TFA |
| I3LUP6 | Putative nucleophosmin 1 variant 1 | TFA |
| I7H541 | Musashi homolog 2 | TFA |
| K7GLT8 | ATP synthase subunit beta | TFA |
| K7GNZ3 | Nascent polypeptide-associated complex subunit alpha | Salt |
| P80015 | Azurocidin | TFA |
| Q2EN81 | ATP synthase subunit O, mitochondrial | TFA |
| Q3YLA6 | Serine/arginine-rich splicing factor 1 | Salt |
| Q4TTS4 | Histone H1.2-like protein | Salt |
| Q53DY5 | Histone H1.3-like protein | Salt |
| Q5S1U1 | Heat shock protein beta-1 | Salt + TFA |
| Q7M2W6 | Alpha-crystallin B chain | TFA |
| Q95339 | ATP synthase subunit f, mitochondrial | TFA |
| Q9GLP1 | Coagulation factor V | TFA |
| Q9MYT8 | ATP synthase subunit e, mitochondrial | TFA |

Supplementary Table 2: Putative actin-binding proteins of *M. hyopneumoniae*

| **Accession** | **Protein Name** | **Surface Exposed** |
| --- | --- | --- |
| ***P97 & P102 adhesins*** | | |
| Q4A925 | Putative adhesin like-protein P146 | + |
| Q4A926 | P135/Mhp683 (P102 paralog) | + |
| Q4A9J1 | Putative p76 membrane protein | + |
| Q4A9J2 | Putative P216 surface protein | + |
| Q4A9W4 | Mhp385 | + |
| Q4A9W5 | Mhp384 | + |
| Q4AA66 | Mhp107 | + |
| Q4AA67 | Mhp108 | + |
| Q4AAD5 | P102 | + |
| Q4AAD6 | P97 Adhesin | + |
| Q4AAM4 | Mhp271 | + |
| Q601C7 | Mhp275 (No J homolog) | - |
| ***Glycolytic enzymes*** | | |
| P0C0J3 | L-lactate dehydrogenase | + |
| Q4A9Q0 | Hexulose-6-phosphate isomerase | - |
| Q4A9J8 | Phosphoglycerate kinase | + |
| Q4A9X8 | Glycerol kinase | - |
| Q4AA88 | Enolase | + |
| Q4AAL7 | pdhB (Pyruvate dehydrogenase E1-beta subunit) | + |
| Q4AAR8 | Glyceraldehyde 3-phosphate | + |
| Q4AAM2 | 6-phosphofructokinase | + |
| ***Lipoproteins*** | | |
| P0C0J8 | 46 kDa surface antigen | + |
| Q4A932 | Putative prolipoprotein P65 | + |
| Q4A9A0 | Pullulanase | - |
| Q4A9X1 | Putative lipoprotein | + |
| ***Translational & chaperone proteins*** | | |
| Q4A9G1 | Elongation factor Tu | + |
| Q4AAQ8 | Elongation factor 4 | - |
| Q4AAR3 | Heat shock protein DnaJ | + |
| Q4AAR4 | Chaperone protein dnaK | + |
| ***Ribosomal proteins*** | | |
| P0C0J9 | 30S ribosomal protein S15 | + |
| Q4A937 | 50S ribosomal protein L13 | - |
| Q4A938 | 30S ribosomal protein S9 | + |
| Q4A943 | 50S ribosomal protein L9 | - |
| Q4A967 | 50S ribosomal protein L10 | - |
| Q4A9B0 | 30S ribosomal protein S4 | + |
| Q4A9M8 | 50S ribosomal protein L11 | - |
| Q4A9M9 | 50S ribosomal protein L1 | + |
| Q4AA43 | 30S ribosomal protein S6 | + |
| Q4AA45 | 30S ribosomal protein S18 | - |
| Q4AA52 | 30S ribosomal protein S16 | - |
| Q4AA54 | 50S ribosomal protein L19 | + |
| Q4AA93 | 30S ribosomal protein S20 | + |
| Q4AAE0 | 50S ribosomal protein L3 | + |
| Q4AAE1 | 50S ribosomal protein L4 | + |
| Q4AAE2 | 50S ribosomal protein L23 | + |
| Q4AAE3 | 50S ribosomal protein L2 | + |
| Q4AAE5 | 50S ribosomal protein L22 | + |
| Q4AAE6 | 30S ribosomal protein S3 | + |
| Q4AAE7 | 50S ribosomal protein L16 | + |
| Q4AAE8 | 50S ribosomal protein L29 | + |
| Q4AAE9 | 30S ribosomal protein S17 | - |
| Q4AAF0 | 50S ribosomal protein L14 | - |
| Q4AAF1 | 50S ribosomal protein L24 | + |
| Q4AAF2 | 50S ribosomal protein L5 | + |
| Q4AAF4 | 30S ribosomal protein S8 | + |
| Q4AAF5 | 50S ribosomal protein L6 | + |
| Q4AAF6 | 50S ribosomal protein L18 | + |
| Q4AAF7 | 30S ribosomal protein S5 | - |
| Q4AAF8 | 50S ribosomal protein L15 | + |
| Q4AAG4 | 30S ribosomal protein S13 | + |
| Q4AAG5 | 30S ribosomal protein S11 | + |
| Q4AAG7 | 50S ribosomal protein L17 | + |
| Q4AAK1 | 50S ribosomal protein L27 | + |
| Q4AAK2 | 50S ribosomal protein L21 | + |
| Q4AAL1 | 50S ribosomal protein L28 | - |
| Q4AAQ5 | 30S ribosomal protein S7 | + |
| Q4AAW8 | 30S ribosomal protein S2 | + |
| ***tRNA proteins*** | | |
| Q4A941 | Leucine--tRNA ligase | - |
| Q4A947 | Cysteine--tRNA ligase | - |
| Q4A952 | Probable tRNA threonylcarbamoyladenosine biosynthesis protein | - |
| Q4A998 | Threonine--tRNA ligase | - |
| Q4A9S1 | Asparagine--tRNA ligase | - |
| Q4A9S6 | Methionyl-tRNA synthetase | - |
| Q4A9V2 | Proline--tRNA ligase | - |
| Q4AA64 | Phenylalanine--tRNA ligase beta subunit | + |
| Q4AA95 | Aspartyl-tRNA synthetase | - |
| Q4AAD3 | Alanine--tRNA ligase | + |
| Q4AAS1 | Isoleucine--tRNA ligase | - |
| Q4AAX3 | Tyrosine--tRNA ligase | - |
| ***ATP- binding proteins*** | | |
| Q4A980 | Putative sugar ABC transporter ATP-binding protein | - |
| Q4A9H3 | Xylose ABC transporter ATP-binding protein | - |
| Q4A9K9 | ATP synthase alpha chain | + |
| Q4A9L9 | Putative ABC transporter ATP-binding protein | - |
| Q4A9N6 | Putative ABC transporter ATP-binding protein | - |
| Q4A995 | Putative ATP-binding protein | + |
| Q4A9V4 | Putative ABC transporter ATP-binding protein | - |
| Q4AA33 | ABC transporter ATP binding protein | - |
| Q4AA74 | Energy-coupling factor transporter ATP-binding protein EcfA 2 | - |
| Q4AA75 | Energy-coupling factor transporter ATP-binding protein EcfA 1 | - |
| Q4AAA5 | Ribose ABC transport ATP-binding protein | - |
| Q4AAS9 | ABC transporter ATP binding protein | - |
| Q4AAC0 | Oligopeptide ABC transporter ATP-binding protein | - |
| Q4AAT0 | Putative ABC transporter ATP-binding protein | - |
| ***Non-Grouped & putative uncharacterised proteins*** | | |
| Q4A931 | Chromosome partition protein | + |
| Q4A951 | Transcription antitermination protein | - |
| Q4A981 | Putative Uncharacterised Protein | + |
| Q4A982 | Inorganic pyrophosphatase | + |
| Q4A9A2 | Translation initiation factor IF-2 | - |
| Q4A9A3 | Ribosome-binding factor A | - |
| Q4A9A8 | DNA glycosylase | - |
| Q4A9E7 | Putative uncharacterized protein | - |
| Q4A9E9 | Uridylate kinase | - |
| Q4A9F7 | Deoxyribose-phosphate aldolase | + |
| Q4A9F8 | Uracil phosphoribosyltransferase | + |
| Q4A9I0 | Acetate kinase | + |
| Q4A9K0 | Putative uncharacterized protein | + |
| Q4A9N3 | FMN-dependent NADH-azoreductase | + |
| Q4A9N9 | Probable GTP-binding protein | - |
| Q4A9Q2 | Putative Uncharacterised Protein | - |
| Q4A9R9 | ATP-dependent helicase PcrA | - |
| Q4A9S4 | Ribonuclease 3 | - |
| Q4A9Y6 | Putative uncharacterized protein (P80) | + |
| Q4AA35 | Glycerophosphoryl diester phosphodiesterase | - |
| Q4AA39 | Putative uncharacterized protein | + |
| Q4AA58 | CTP synthetase | - |
| Q4AA61 | Putative Uncharacterised Protein | - |
| Q4AA76 | Hypoxanthine phosphoribosyltransferase | + |
| Q4AAB5 | Ribonucleoside-diphosphate reductase | - |
| Q4AAB8 | Putative Uncharacterised Protein | + |
| Q4AAG0 | Adenylate kinase | - |
| Q4AAJ3 | Glutamate--tRNA ligase | - |
| Q4AAJ6 | Putative uncharacterised protein | + |
| Q4AAK7 | Pyruvate Kinase | + |
| Q4AAK8 | Translation initiation factor IF-3 | + |
| Q4AAL9 | Adenine phosphoribosyltransferase | + |
| Q4AAM3 | DNA gyrase subunit B | + |
| Q4AAM9 | ATP-dependent protease binding protein | - |
| Q4AAN0 | Putative Uncharacterised Protein | + |
| Q4AAN3 | Prolipoprotein diacylglyceryl transferase | - |
| Q4AAN4 | Thiol peroxidase | + |
| Q4AAP5 | Protein translocase subunit SecA | - |
| Q4AAP7 | Purine-nucleoside phosphorylase | + |
| Q4AAP8 | Thymidine phosphorylase | + |
| Q4AAP9 | NADH Oxidase | + |
| Q4AAR0 | Bacterial nucleoid DNA-binding protein | + |
| Q4AAR9 | Topoisomerase IV subunit B | - |
| Q4AAT9 | Heat-inducible transcription repressor HrcA | - |
| Q4AAU7 | DNA polymerase III beta subunit | - |
| Q4AAV6 | Protein RecA | - |
| Q4AAV7 | ATP synthase subunit beta | + |
| Q4AAW5 | Glycyl-tRNA synthetase | - |
| Q4AAW6 | Signal recognition particle protein | + |
| Q4AAX2 | Putative uncharacterized protein | - |
| Q600K9 | Mhp446 | - |
